# Biosynthesis and import of the cytoprotective extremolytes ectoine and hydroxyectoine in the phylum *Planctomycetota*

**DOI:** 10.3389/fmicb.2026.1823765

**Published:** 2026-06-24

**Authors:** Laura Czech, Nicolai Kallscheuer, Sandra Wiegand, Marcus Lechner, Christian Jogler, Erhard Bremer

**Affiliations:** 1 Center for Synthetic Microbiology (SYNMIKRO), Marburg University, Marburg, Germany; 2 Faculty of Chemistry, Marburg University, Marburg, Germany; 3 Department of Microbial Interactions, Institute of Microbiology, Friedrich-Schiller-University, Jena, Germany; 4 Department of Microbiology, Radboud University, Nijmegen, Netherlands; 5 Cluster of Excellence Balance of the Microverse, Friedrich-Schiller-University, Jena, Germany; 6 Faculty of Biology, Marburg University, Marburg, Germany

**Keywords:** ectoine, extremolytes, genome mining, osmotic stress, transporters

## Abstract

Many bacteria inhabiting high-salinity environments accumulate compatible solutes, water-soluble organic compounds that are highly congruent with cellular biochemistry and physiology. This “salt-out” strategy counteracts cytoplasmic water loss, maintains turgor within physiologically acceptable limits, and enables growth under conditions conferring osmotic stress. Ectoine and its derivative hydroxyectoine are prominent compatible solutes that function not only as efficient osmotic stress protectants but also serve as chemical chaperones and cytoprotectants. These properties have driven industrial-scale biotechnological production of ectoines and their broad practical applications. Despite their well-established role in stress protection, the distribution of ectoines within the widespread *Planctomycetota* has remained largely unexplored. Here, we analyzed the genomes of 163 type strains within this phylum and identified 23 species harboring ectoine biosynthetic gene clusters (*ect*). These clusters were predominantly present in marine-, saline- and brackish water-associated members of the families *Planctomycetaceae* and *Pirellulaceae,* with few representatives present in *Lacipirellulaceae*. Experimental validation of *ect* cluster functionality in four *Planctomycetota* species confirmed increased ectoine and hydroxyectoine production under osmotic stress, supporting their role as extremolytes. All *ect* clusters co-localize with genes encoding compatible solute transporters from the ABC (EhuABCD), TRAP-T (TeaABC; UheABC), or sodium solute symporter (SSS; EctI) families. Functional characterization of EctI from the type strain *Rubinisphaera brasiliensis* demonstrated uptake of hydroxyectoine and additional compatible solutes, including proline betaine, homobetaine, glycine betaine, and dimethylsulfoniopropionate (DMSP). Together, our findings reveal a lineage-specific adaptation of *Planctomycetota* to sustained osmotic stress through both synthesis and uptake of ectoines, providing new insights into the ecophysiology of these microorganisms.

## 1 Introduction

*Planctomycetota* (formerly *Planctomycetes*) is a phylum within the *Planctomycetota –Verrucomicrobiota – Chlamydiota* (PVC) superphylum of the domain *Bacteria* (Wagner and Horn, 2006). Since their discovery, these microorganisms have attracted attention for their unusual cellular organization, distinctive non-FtsZ-based budding division, diverse lifestyles, ecological niches, metabolism, contributions to global nutrient cycles, and biotechnological potential (Kündgen et al., 2025; Kallscheuer et al., 2024; Odelgard et al., 2024; Wiegand et al., 2018; Kallscheuer and Jogler, 2021; Boedeker et al., 2017; Lage et al., 2025; Wurzbacher et al., 2024; Shiratori et al., 2019). *Planctomycetota* continue to intrigue taxonomists, physiologists, and molecular biologists alike (Wiegand et al., 2018; Lage et al., 2025; Devos et al., 2020).

Although initially difficult to cultivate, recent advances have enabled the isolation of numerous novel species (Kündgen et al., 2025; Wiegand et al., 2018; Lage et al., 2025; Devos et al., 2020; Wiegand et al., 2020; Vitorino and Lage, 2022; Dedysh et al., 2020; Dedysh et al., 2021). In addition, the growing availability of genome and meta-genome sequences has facilitated detailed taxonomic, bioinformatic, and experimental analyses of their lifestyle and metabolic capabilities (Kündgen et al., 2025; Kallscheuer and Jogler, 2021; Lage et al., 2025; Hägglund et al., 2026). *Planctomycetota* are widespread in freshwater and brackish water habitats, marine environments, deep-sea sediments, hydrothermal vents, limnic environments, terrestrial ecosystems, and plant- and algae-associated habitats. Metagenomic studies underscore the global ecological significance of these microorganisms (Wiegand et al., 2018; Kallscheuer and Jogler, 2021; Lage et al., 2025; Bengtsson and Ovreas, 2010; Kallscheuer et al., 2021; Godinho et al., 2021). Their metabolic activities contribute to worldwide operating carbon, nitrogen, and sulfur cycles, including the eco-physiological very important anaerobic ammonium oxidation process (anammox) allowing the removal of fixed nitrogen from the environment and waste waters (Wiegand et al., 2018; Lage et al., 2025; Hägglund et al., 2026; Suarez et al., 2023; Lenferink et al., 2024; Klimek et al., 2024).

Many *Planctomycetota* encounter sudden or sustained increases in salinity or osmolarity in their diverse ecological niches. Genomic analyses indicate that these organisms generally do not rely on the “salt-in” strategy (Galinski and Trüper, 1994; Gunde-Cimerman et al., 2018), which involves the long-term accumulation of inorganic ions (e.g., K^+^ and Cl^−^) accompanied by evolutionary adaptations across the entire proteome that leaves an acid footprint on most proteins (Gunde-Cimerman et al., 2018; Roesser and Müller, 2001). Instead, they appear to rely on the “salt-out” strategy (Galinski and Trüper, 1994; Gunde-Cimerman et al., 2018; Kempf and Bremer, 1998) in which compatible solutes, highly water-soluble organic molecules that remain congruent with cellular biochemistry and physiology even at very high cytoplasmic concentrations, are accumulated. The accumulation of these types of solutes (da Costa et al., 1998), whether newly synthesized or imported from the environment, is tightly regulated, linked to the osmotic stress imposed onto cells, prevents a sustained high ionic cytoplasm, and ultimately supports growth under osmotically unfavorable conditions (Bremer and Krämer, 2019; Wood, 2011; Hoffmann and Bremer, 2017; van den Berg et al., 2017). Prior studies have reported the synthesis of various types of compatible solutes in members of the *Planctomycetota*, including *Gimesia maris*, *Rubinisphaera brasiliensis*, *Rhodopirellula baltica*, and *Natronomicrosphaera hydrolytica* (d'Avo et al., 2013; Cunha et al., 2013; Ferreira et al., 2016; Schwibbert et al., 2011; Jeske et al., 2013; Wecker et al., 2009; Sorokin et al., 2025). Observed or predicted compatible solutes include sucrose, trehalose, *α*-glutamate, glucosylglycerate, mannosyl-(1,2)-glucosylglycerate, the peptide *N*-acetylglutaminylglutamine amide, and ectoines.

Our study focuses specifically on ectoine (Galinski and Pfeiffer, 1985) and its hydroxylated derivative 5-hydroxyectoine (Inbar and Lapidot, 1988), two of the most widely synthesized compatible solutes by bacteria (Czech et al., 2018; Hermann et al., 2020; Kunte et al., 2014; Pastor et al., 2010). Beyond osmotic protection, ectoines act as broad-spectrum cytoprotectants, stabilizing cells against high and low temperature extremes and desiccation, and serve as chemical chaperones for membranes, nucleic acids, and proteins (Knapp et al., 1999; Tanne et al., 2014; Kurz, 2008; Bursy et al., 2008; Kuhlmann et al., 2008; Ma et al., 2017). Ectoine-conferred cytoprotective properties have made them commercially valuable for a wide range of practical applications, particularly in skin care (Czech et al., 2018; Kunte et al., 2014; Pastor et al., 2010; Becker and Wittmann, 2020; Ferreira et al., 2025), with large-scale industrial production processes already well established (Kunte et al., 2014; Becker and Wittmann, 2020; Ferreira et al., 2025; Lentzen and Schwarz, 2006; Wang et al., 2025).

**Figure 1:**
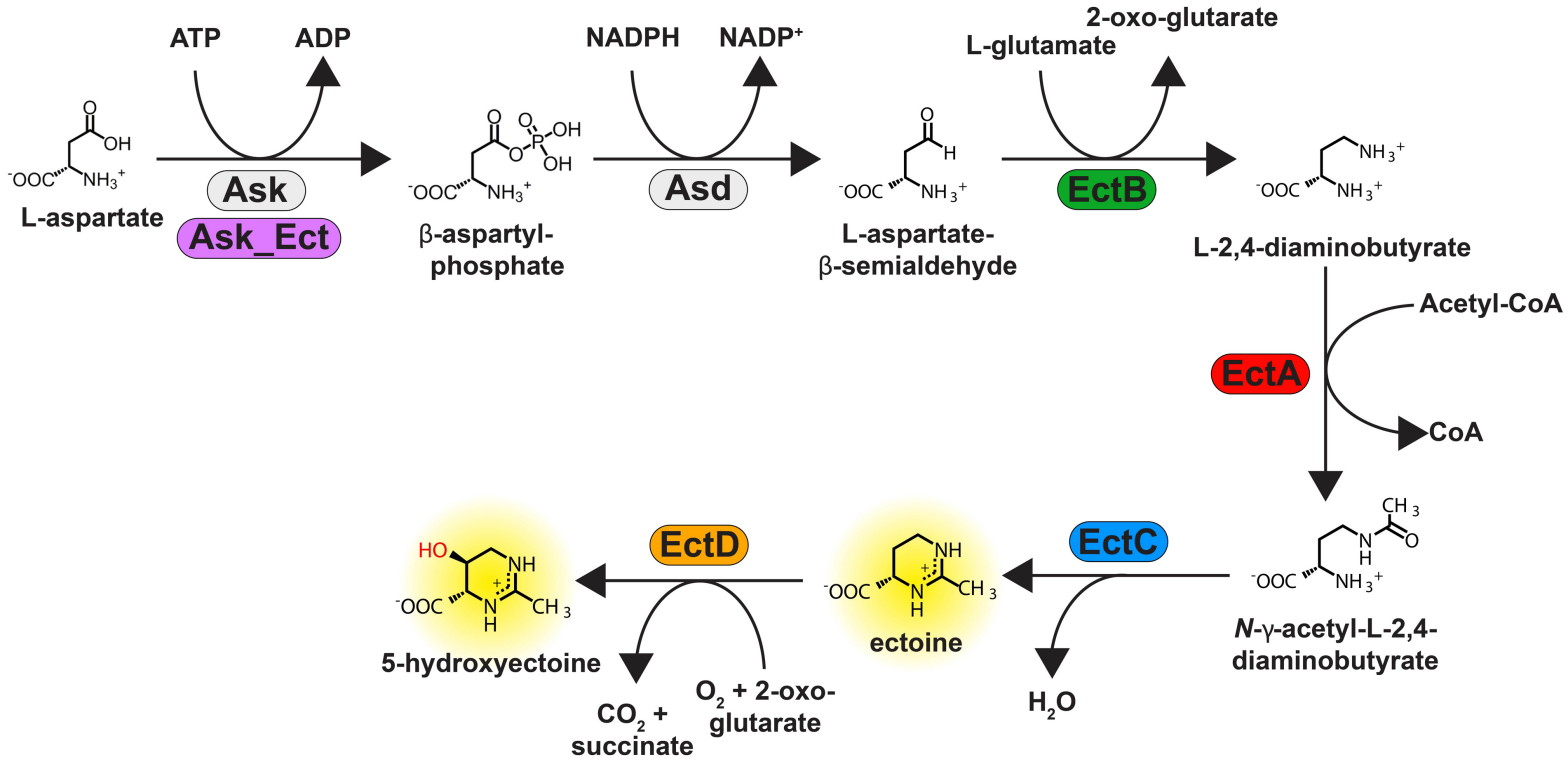
Biosynthetic route for ectoine and its derivative 5-hydroxyectoine. These data were compiled from the literature (Czech et al., 2018; Hermann et al., 2020; Kunte et al., 2014; Pastor et al., 2010; Ono et al., 1999; Peters et al., 1990). Structural analysis of the EctB, EctA, EctC, and EctD ectoine/hydroxyectoine biosynthetic enzymes (Hillier et al., 2020; Czech et al., 2019; Höppner et al., 2014; Richter et al., 2019; Stöveken et al., 2011), and biochemistry of the specialized aspartokinase Ask_Ect (Stöveken et al., 2011) have been reported.

Substantial advances in the biochemistry and structural biology of the ectoine and hydroxyectoine biosynthetic routes have been made since their original discoveries (Hermann et al., 2020; Ono et al., 1999; Peters et al., 1990). Ectoine synthesis begins from L-aspartate-*β*-semialdehyde, a central metabolic intermediate, and proceeds sequentially through the activities of three enzymes to generate the cyclic ectoine molecule (Figure 1). These biosynthetic enzymes are the L-2,4-diaminobutyrate transaminase EctB (EC 2.6.1.76), the L-2,4-diaminobutyrate acetyltransferase EctA (EC 2.3.1.178), and the ectoine synthase EctC (EC 4.2.1.108), the signature enzyme of the ectoine biosynthetic route (Ono et al., 1999; Peters et al., 1990). In many ectoine-producing organisms, ectoine can be converted into 5-hydroxyectoine with high positional and stereochemical specificity by the ectoine hydroxylase EctD (EC 1.14.11.55), thereby conferring additional cytoprotective properties on this ectoine derivative (Bursy et al., 2007; Argandona et al., 2021). Crystal structures of all four ectoine/hydroxyectoine biosynthetic enzymes have now been established (Hermann et al., 2020; Richter et al., 2020; Hillier et al., 2020; Czech et al., 2019; Höppner et al., 2014).

Ectoine biosynthetic genes are typically organized in an *ectABC* operon, often accompanied by *ectD* (Schwibbert et al., 2011; Czech et al., 2018; Hermann et al., 2020; Pastor et al., 2010; Czech et al., 2022). In many microorganisms, these genes co-localize with additional loci involved in precursor supply (e.g., *ask_ect* and *asd*), transport systems (import/export) for ectoines, and MscS-type mechanosensitive channels for the rapid release of solutes upon sudden osmotic down-shocks to preserve cellular integrity (Czech et al., 2022; Booth, 2014). The roles of ectoines as osmotic stress and cytoprotectants are reflected in the transcriptional induction of *ect* biosynthetic genes by increased salinity or osmolarity (Bursy et al., 2008; Ma et al., 2017; Bursy et al., 2007; Argandona et al., 2021; Czech et al., 2022; Czech et al., 2018; Calderon et al., 2004), by extremely high or low growth temperatures, or during stationary phase (Bursy et al., 2008; Kuhlmann et al., 2008; Ma et al., 2017). A MarR-type regulator, in the literature referred to as EctR or CosR, functions as a transcriptional repressor (Mustakhimov et al., 2010; Gregory et al., 2020; Shikuma et al., 2013); the encoding gene is frequently located adjacent to *ect* gene clusters. However, the physiological cues governing its DNA-binding activity to *ect* promoter regions remains largely unresolved (Mustakhimov et al., 2010; Shikuma et al., 2013).

With respect to studies on compatible solutes produced by *Planctomycetota* (d'Avo et al., 2013; Cunha et al., 2013; Ferreira et al., 2016; Jeske et al., 2013; Wecker et al., 2009), the biochemistry underlying the biosynthesis of glucosylglycerate and mannosyl-(1,2)-glucosylglycerate has been experimentally characterized in some detail in *R. baltica* (d'Avo et al., 2013; Cunha et al., 2013). However, since the initial detection of ectoines in osmotically stressed cultures of *G. maris* (basonym: *Planctomyces maris*) and *R. brasiliensis* (Ferreira et al., 2016), together with *in silico* identification of ectoine biosynthetic genes in the genome of *Blastopirellula marina* DSM 3645 (Schwibbert et al., 2011), no comprehensive molecular or physiological investigations have studied either the distribution of ectoines or their osmotically regulated biosynthesis within the phylum *Planctomycetota*. Given the broad ecological distribution of strains belonging to this large phylum (Kallscheuer et al., 2024; Odelgard et al., 2024; Wiegand et al., 2018; Kallscheuer and Jogler, 2021; Lage et al., 2025), osmotic challenges encountered across diverse habitats, and the well-established role of ectoines as extremolytes with significant biotechnological applications (Czech et al., 2018; Kunte et al., 2014; Pastor et al., 2010; Becker and Wittmann, 2020; Ferreira et al., 2025; Lentzen and Schwarz, 2006; Wang et al., 2025), we systematically investigated the prevalence of ectoine biosynthetic gene clusters (*ect*) within this group of microorganisms. In this study, we analyzed genome sequences from the entire currently available collection of 163 type strains of *Planctomycetota*. Focusing on type strains rather than on the rapidly growing collection of *Planctomycetota* metagenomes ensured analytical stringency and provided reliable ecological context based on the original isolation habitats of the respective species. In addition, the availability of these strains through publicly accessible culture collections facilitates future experimental validation and further investigation.

We found that the genomes of approximately 14% of the 163 type strains of *Planctomycetota* harbor *ect* biosynthetic genes, all of which co-localize with genes encoding various types of putative ectoine import systems. Overall, our findings reveal a lineage-specific adaptation in *Planctomycetota*, potentially shaped by horizontal gene transfer, in which the synthesis and uptake of ectoine and hydroxyectoine contribute to enhanced tolerance to osmotic stress. These insights deepen our understanding of the ecophysiology of *Planctomycetota* and further underscore their potential for biotechnological applications.

## 2 Materials and methods

### 2.1 Chemicals and reagents

Ectoine and hydroxyectoine were kindly provided by the bitop AG (Dortmund, Germany). All other compatible solutes used in this study were taken from laboratory stocks; their sources have previously been detailed (Holtmann and Bremer, 2004; Hoffmann and Bremer, 2011). Acetonitrile (HPLC-grade) was obtained from VWR International GmbH (Darmstadt, Germany). Ampicillin, and all other chemicals were purchased from Sigma-Aldrich (Steinheim, Germany), Serva Electrophoresis GmbH (Heidelberg, Germany), or Carl Roth GmbH (Karlsruhe, Germany). Enzymes for DNA manipulations were obtained from Thermo Fisher Scientific GmbH (Dreieich, Germany), Roche Diagnostics GmbH (Mannheim, Germany) or New England BioLabs GmbH (Frankfurt, Germany).

### 2.2 Recombinant DNA procedures and construction of plasmids

Routine manipulations of plasmid DNA, the construction of recombinant plasmids, and the isolation of plasmid DNA from *Escherichia coli* were carried out using standard techniques. The *R. brasiliensis ectI* gene (NCBI genome accession number: CP002546.1; NCBI protein accession number: WP_013629616.1) was synthesized and codon-optimized for expression in *E. coli* by GeneArt Gene Synthesis (Thermo Fisher Scientific, Dreieich, Germany). The DNA-sequence of the codon-optimized *R. brasiliensis ectI* gene was deposited in the NCBI Genbank under accession number ON778465. The recombinant *ectI* gene was amplified by PCR from a plasmid DNA provided by GeneArt using custom synthesized primers LC138_Rubi_SSS_for (AAACCATGGATAGCGTTATTGTTCTGGCCAGC) and LC139_Rubi_SSS_rev (AAAAAGCTTTTAGCTTTCTTGCTGGCTATTGCTTG). DNA-primers were purchased from Sigma Aldrich (Steinheim, Germany). Appropriate DNA-sequences with unique restriction enzyme recognition sites (for NcoI and HindIII) were added to the 5′ ends of the DNA-primers used to synthesize the PCR product (corresponding restriction sites are underlined in the DNA-sequence of the primers as shown above). The obtained PCR product was digested with NcoI and HindIII and the resulting DNA-fragment was then cloned into the expression vector pTrc99a (Amann et al., 1988). This positioned the transcription of the recombinant *ectI* gene from *R. brasiliensis* under the control of the LacI/IPTG controlled *lac* promoter carried on the pTrc99a vector (Amann et al., 1988) and resulted in the assembly of plasmid pLC211.

### 2.3 Bacterial strains, media and growth conditions

Four *Planctomycetota* species were used to experimentally assess the *in silico* predicted synthesis of ectoines. *Rubinisphaera italica* Pan54 (DSM 29369) (Kallscheuer et al., 2020), *Polystyrenella longa* Pla110 (DSM 103387) (Peeters et al., 2020), *Bremerella volcania* Pan97 (DSM 101992) (Rensink et al., 2020), and *Blastopirellula retiformator* Enr8 (DSM 100415) (Kallscheuer et al., 2020).

The cultivation of all four strains was performed in a chemically defined minimal medium supplemented with different concentrations of NaCl to increase the salinity/osmolarity of the growth medium and thereby trigger the synthesis of ectoines. For the preparation of growth media, stock solutions of artificial sea water without sodium chloride (ASW-NaCl), the trace element solution SL10, a vitamin solution, metal salt solution 44 and Hutner’s basal salt solution were prepared. The medium 2xASW-NaCl contained 7.84 g L^−1^ Na_2_SO_4_, 21.28 g L^−1^ MgCl_2_ × 6 H_2_O, 2.86 g L^−1^ CaCl_2_ × 2 H_2_O, 0.384 g L^−1^ NaHCO_3_, 1.384 g L^−1^ KCl, 0.192 g L^−1^ KBr, 0.052 g L^−1^ H_3_BO_3_, 0.08 g L^−1^ SrCl_2_ × 6 H_2_O, and 0.006 g L^−1^ NaF. It was always prepared fresh, as the stock solution was not filtered or autoclaved. The SL10 trace element solution was prepared with 1.5 g L^−1^ Na-nitrilotriacetate, 500 mg L^−1^ MnSO_4_ × H_2_O, 100 mg L^−1^ FeSO_4_ × 7 H_2_O, 100 mg L^−1^ Co(NO_3_)_2_ × 6 H_2_O, 100 mg L^−1^ ZnCl_2_, 50 mg L^−1^ NiCl_2_ × 6 H_2_O, 50 mg L^−1^ H_2_SeO_3_, 10 mg L^−1^ CuSO_4_ × 5 H_2_O, 10 mg L^−1^ AlK(SO_4_)_2_ × 12 H_2_O, 10 mg L^−1^ H3BO_3_, 10 mg L^−1^ NaMoO_4_ × 2 H_2_O and 10 mg L^−1^ Na_2_WO_4_ × 2 H_2_O. The solution was sterilized by filtration and stored in the dark at 4 °C. The vitamin solution contained 10 mg L^−1^ *p*-aminobenzoic acid, 4 mg L^−1^ biotin, 20 mg L^−1^ pyridoxine hydrochloride, 10 mg L^−1^ thiamine hydrochloride, 10 mg L^−1^ calcium-pantothenate, 4 mg L^−1^ folic acid, 10 mg riboflavin, 10 mg L^−1^ nicotinamide and 0.2 mg L^−1^ vitamin B12. *p*-Aminobenzoic acid was dissolved first. The solution was sterilized by filtration and stored in the dark at 4 °C. Metal salt solution 44 consisted of 250 mg L^−1^ Na_2_-EDTA, 1095 mg L^−1^ ZnSO_4_ × 7 H_2_O, 500 mg L^−1^ FeSO_4_ × 7 H_2_O, 154 mg L^−1^ MnSO_4_ × H_2_O, 39.5 mg L^−1^ CuSO_4_ × 5 H_2_O, 20.3 mg L^−1^ CoCl_2_ × 6 H_2_O and 17.7 mg L^−1^ Na_2_B_4_O_7_ × 10 H_2_O. EDTA was dissolved by adding a few drops of concentrated H_2_SO_4_ to retard the precipitation of heavy metal ions. The solution was sterilized by filtration and stored at 4 °C. Hutner’s basal salt solution was prepared with 10 g L^−1^ nitrilotriacetic acid (NTA), 29.7 g L^−1^ MgSO_4_ × 7 H_2_O, 3.34 g L^−1^ CaCl_2_ × 2 H_2_O, 0.01267 g L^−1^ Na_2_MoO_4_ × 2 H_2_O, 0.099 g L^−1^ FeSO_4_ × 7 H_2_O and 50 mL L^−1^ metal salt solution 44. NTA was dissolved in 700 mL distilled water by adjusting the pH to 7.2 with KOH. All further components were dissolved separately and added slowly to the basal solution. The solution was sterilized by filtration and stored at 4° C.

The final growth medium contained 250 mL L^−1^ 2 × ASW-NaCl, 20 mL L^−1^ Hutner’s basal salt solution and 2.38 g L^−1^ HEPES as buffer. The used molar concentrations of NaCl were 0 mM, 100 mM, 350 mM, 500 mM, 700 mM, 900 mM, 1,200 mM, or 1,600 mM. These concentrations were reached by adding 0 mL, 20 L^−1^, 70 mL L^−1^, 100 mL L^−1^, 140 mL L^−1^, 180 mL L^−1^, 240 mL L^−1^, or 320 mL L^−1^, of a 5 M NaCl solution (292.2 g L^−1^) to the growth medium, respectively. The volume was adjusted to 960 mL with distilled water, and the pH was adjusted to 7.0 before the media were autoclaved. Finally, the medium was complemented with 1.5 g L^−1^ glucose, 1.5 g L^−1^ *N*-acetylglucosamine, 5 mL vitamin solution and 1 mL SL10.

The concentration of NaCl added to the growth medium was dependent on the species under study; it is specified in the description of the individual experiments. For growth experiments, the various strains were precultured in medium containing 250 mM NaCl; the pre-culture cells were washed before they were used for the inoculation of the main cultures. *R. italica* Pan54, *B. retiformator* Enr8, and *B. volcania* Pan97 were grown at 28 °C, while *P. longa* Pla110 was incubated at room temperature. The optical densities at 600 nm (OD_600_) of the various cultures were measured once a day over the course of 16 days, or until the stationary phase of the culture was reached. Subsequently, the cells were harvested by centrifugation and stored at −20 °C for the quantification of their ectoine/hydroxyectoine content by HPLC analysis.

The *E. coli* K-12 wild type laboratory strain MC4100 (Ferenci et al., 2009) is the parent of strain MKH13 [*Δ*(*proP*)2 Δ(*proU:spc*)608 (Spc^r^)] (Haardt et al., 1995) carrying defects in the genes for the osmotically inducible compatible solute uptake systems ProP and ProU, and is unable to synthesize glycine betaine from imported choline (Lucht and Bremer, 1994). The ProP and ProU transport systems possess a broad substrate specificity (Haardt et al., 1995; Lucht and Bremer, 1994; MacMillan et al., 1999), which also includes ectoine and hydroxyectoine (MacMillan et al., 1999; Jebbar et al., 1992). *E. coli* strains were routinely maintained on Luria Bertani (LB) agar plates and propagated in liquid LB medium (Miller, 1972). When they contained a recombinant plasmid, ampicillin (100 μg mL^−1^) was added to the growth medium. For the functional assessment of a plasmid-encoded gene for the *R. brasiliensis* EctI-type importer (Czech et al., 2022), the corresponding *E. coli* strains were grown in minimal medium A (MMA) supplemented with 0.2% (w/v) glucose as the carbon source, 1 mM MgSO4, and 1.5 μM thiamine (Miller, 1972). The osmolarity of the MMA growth medium was increased by adding NaCl to a concentration specified in individual growth experiments. Shake-flask cultures were incubated at 37 °C in a shaking water bath set to 220 rpm.

### 2.4 Osmotic stress protection assays

Osmotic stress protection assays with the *E. coli* strain MKH13 (Czech et al., 2022; Haardt et al., 1995; Kempf and Bremer, 1995) carrying either the empty vector pTrc99a (Amann et al., 1988) or the pTrc99a-derived plasmid pLC211 (encoding the codon-optimized *ectI* gene from *R. brasiliensis*) were conducted in 48-well plates. The pre-cultures were prepared in 100-mL Erlenmeyer flasks (culture volume of 20 mL) by growing the cells (at 37 °C) in MMA containing either no additional NaCl or 0.3 M additional NaCl to pre-adapt the cells to the increased salinity before they were used to inoculate main cultures which contained 0.8 M NaCl. This concentration of NaCl severely restricts the growth of strain MKH13 and its derivatives in the absence of externally supplied compatible solutes unless the genes for a functional compatible solute transporter are provided on a plasmid and appropriate substrates are present in the growth medium (Czech et al., 2022; Haardt et al., 1995). Main cultures were inoculated in 48-well plates to an OD578 of 0.05 and grown in MMA containing 0 M NaCl or 0.8 M NaCl in the absence or in the presence of the various compatible solutes (1 mM final concentration). Each well contained 500 μL of medium and the well plate was incubated in an Epoch 2 microplate spectrophotometer (Biotek, Bad Friedrichshall, Germany) at 37 °C with double orbital vigorous shaking. Growth of the cultures was monitored by measuring OD578 values every hour for 48 h.

### 2.5 HPLC analysis of ectoine and hydroxyectoine content of selected *Planctomycetota*

The following *Planctomycetota* were grown in chemically defined media containing various concentrations of NaCl: *R. italica* Pan54, *P. longa* Pla110, *B. volcania* Pan97 and *B. retiformator* Enr8. Following the protocol of Bligh and Dyer (Bligh and Dyer, 1959) with minor modifications, low molecular weight organic compounds were extracted with 20% (v/v) ethanol from cell pellets of cultures of these strains by resuspending them in 1 mL 20% (v/v) ethanol, and the mixture was then shaken for 1 h at room temperature. After centrifugation at (16,000 × g at 4 °C for 30 min) to remove cell debris, the ethanolic extracts were transferred into fresh Eppendorf tubes, and ethanol was removed by evaporation (at 65 °C for 20 h). The resulting dried material was re-suspended in 100 μL of distilled water and insoluble material was removed by centrifugation (16,000 × g at 4 °C for 30 min). The extracted samples and the cell-free culture supernatants were diluted ten-fold with distilled water and acetonitrile the final concentration of acetonitrile was 50% (v/v) and were then analyzed for their ectoine/hydroxyectoine content by isocratic high-performance liquid chromatography (HPLC) as described (Czech et al., 2018; Kuhlmann and Bremer, 2002). For these measurements, an Agilent 1260 Infinity LC system (Agilent, Waldbronn, Germany) and a GROM-SIL Amino 1PR column (Dr. Maisch GmbH, Ammerbuch-Entringen, Germany) were originally employed (Kuhlmann and Bremer, 2002). For the current study, a 1260 Infinity Diode Array Detector (DAD) (Agilent) was used instead of the previously employed UV/Vis detector system (Kuhlmann and Bremer, 2002) to detect ectoine and hydroxyectoine as described (Czech et al., 2018). The ectoine and hydroxyectoine content of samples was quantified using the OpenLAB software suite (Agilent, Waldbronn, Germany). Standard curves for the calculation of the ectoine and hydroxyectoine concentrations were determined with commercially available reference samples (obtained from bitop AG, Dortmund, Germany).

### 2.6 Database searches for ectoine/hydroxyectoine biosynthetic gene clusters

For the identification of biosynthetic gene clusters antiSMASH v.8.0 (Blin et al., 2025) was used in relaxed mode and with all extra features activated. The analysis included the 163 RefSeq-annotated reference genomes (Goldfarb et al., 2025) belonging to the phylum *Planctomycetota* (as of April 2026, Supplementary Table S1). Ectoine biosynthetic gene clusters were exported and analyzed with Clinker (Gilchrist, 2021) with a minimum alignment sequence identity of 30%.

### 2.7 Phylogenomics of *Planctomycetota* containing ectoine/hydroxyectoine biosynthetic gene clusters

Phylogenetic tree reconstruction of the current phylum *Planctomycetota* was performed based on the NCBI reference genomes (163 reference genomes as of April 2026) using multi-locus sequence analysis (MLSA) (Alanjary et al., 2019). NCBI RefSeq accession numbers of the used genomes are provided in Supplementary Table S1. The genome sequences of *Verrucomicrobium spinosum* DSM 4136 (RefSeq accession number GCF_000172155.1)*, Kiritimatiella glycovorans* L21-Fru-ABT (RefSeq accession number GCF_001017655.1) and *Lentisphaera araneosa* HTCC 2155 (RefSeq accession number GCF_000170755.1) are members of the *Planctomycetota-Verrucomicrobiota-Chlamydiota* (PVC) superphylum outside of the phylum *Planctomycetota* and served as outgroup. The MLSA-based phylogeny was computed with autoMLST (automlst-simplified-wrapper tool) based on 30 single copy gene-encoded proteins with 1,000 bootstrap replicates (Alanjary et al., 2019). The obtained phylogenetic trees was visualized with iTOL v6 (Letunic and Bork, 2024).

### 2.8 Protein amino acid sequence alignments and modelling of putative ectoine transporter proteins

The sequences of planctomycetotal EctC, EctD, EctR, EhuB, TeaA and EctI proteins were exported from the GenBank files of ectoine biosynthetic gene clusters obtained from antiSMASH (Blin et al., 2025). Characterized reference protein sequences from different bacteria were downloaded under the NCBI protein accession numbers listed in the respective alignments. Protein sequence alignments were performed using the Clustal Omega web application on the EMBL-EBI website (https://www.ebi.ac.uk/jdispatcher/msa/clustalo). Alignments were performed with the pre-set default parameters provided on the website, only the ORDER option was set to “input” instead of “aligned” to align all proteins against the selected reference sequence (that was positioned as the first entry in the protein multi-fasta file). For visualization, the resulting alignments were sent to Mview 1.67 by following the available website link.

Structural models of the EhuB-, and TeaA ectoine/hydroxyectoine substrate binding protein homologs (Kuhlmann et al., 2008; Hanekop et al., 2007), were built using Alphafold2 (Mirdita et al., 2022) and SWISS Model (Waterhouse et al., 2018). Structures of proteins were visualized and analyzed using the PyMol Molecular Graphics System suit (https://www.pymol.org) (Delano, 2002).

## 3 Results

### 3.1 Distribution of ectoine/hydroxyectoine biosynthetic gene clusters in the genomes of *Planctomycetota* type strains

**Figure 2:**
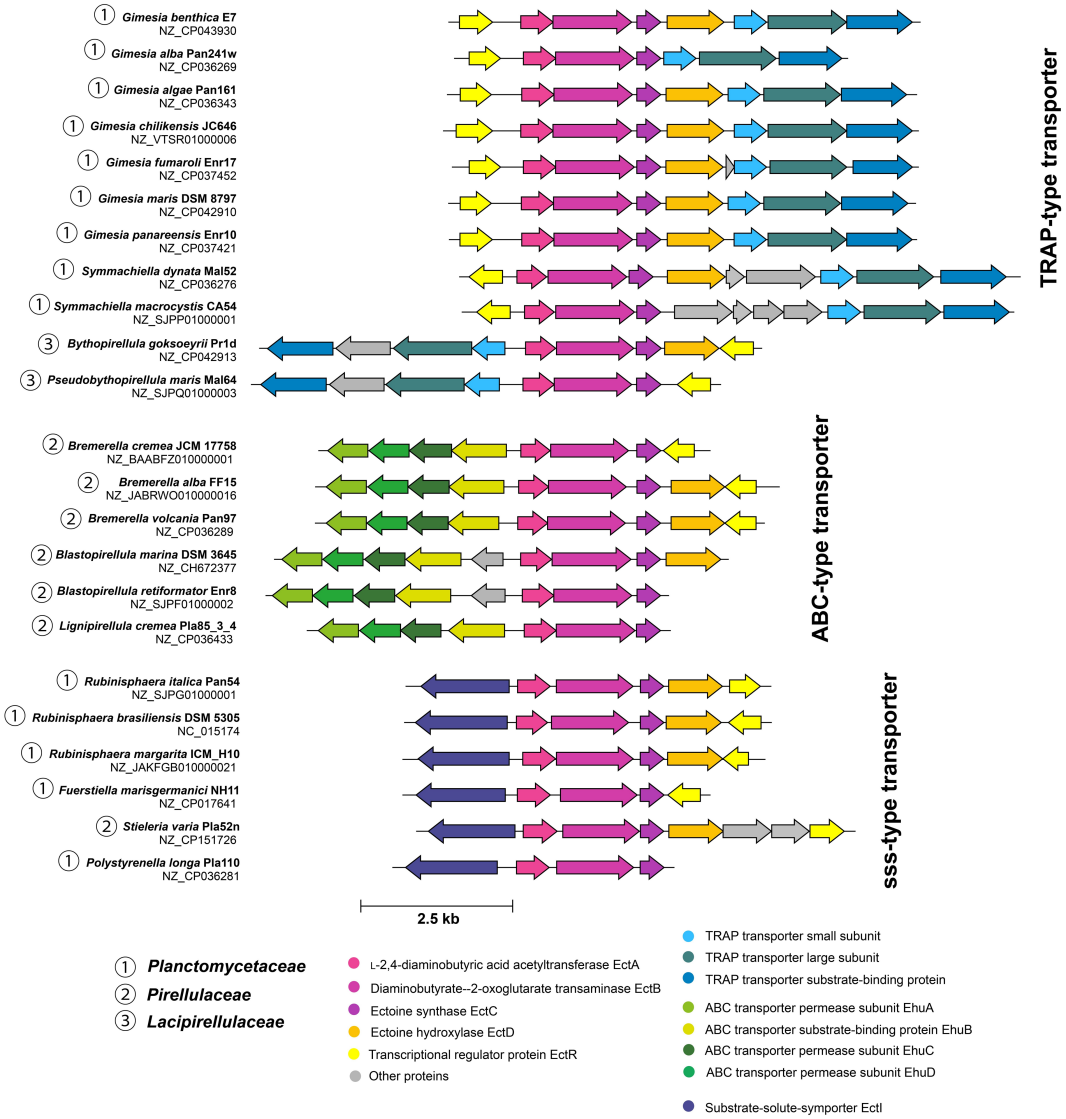
Representative *ect bio*synthetic gene clusters in type strains of *Planctomycetota*. Gene clusters are grouped by associated ectoine/hydroxyectoine transporter types: group I – TRAP-type (TeaABC/UehABC) (Kuhlmann et al., 2008; Lecher et al., 2009); group II – ABC-type (EhuABCD) (Hanekop et al., 2007); group III – SSS-type (EctI) (Czech et al., 2022). *ectABC: ec*toine biosynthetic genes; *ectD: ec*toine hydroxylase; *ectR: Ma*rR-type transcriptional repressor (Mustakhimov et al., 2010). The genetic organization of the ectoine/hydroxyectoine biosynthetic and associated transport genes was visualized with Clinker (Gilchrist, 2021).

To identify ectoine/hydroxyectoine biosynthetic gene clusters, we used antiSMASH v.8.0 (Blin et al., 2025) to analyze the 163 RefSeq-annotated reference genomes (Goldfarb et al., 2025) of the type strains of all species belonging to the current phylum *Planctomycetota* as of April 2026. The retrieved gene clusters were analyzed with Clinker (Gilchrist, 2021). We then focused our analysis on the presence of genes coding for the ectoine synthase EctC, the established signature enzyme of ectoine biosynthesis (Ono et al., 1999; Peters et al., 1990). Using the amino acid sequence of the biochemically and structurally characterized EctC protein from *Paenibacillus lautus* (Czech et al., 2019; Andrys-Olek et al., 2024) as the search query, we identified 23 genomes of *Planctomycetota* encoding an *ectC*-type gene. Subsequent analysis showed that in all cases, *ectA-* and *ectB-*type genes, encoding the enzymes that catalyze the steps preceding ectoine synthase enzyme activity (Richter et al., 2020; Hillier et al., 2020; Richter et al., 2019), were located immediately adjacent to *ectC* on the chromosome, forming a canonical *ectABC* operon-like genetic configuration (Figure 2). This genetic arrangement mirrors the organization observed in many other microorganisms (Schwibbert et al., 2011; Czech et al., 2018; Hermann et al., 2020; Pastor et al., 2010; Imhoff et al., 2020; Reshetnikov et al., 2011; Leon et al., 2018). In 15 genomes with *ectABC* gene clusters, we also detected an aligned *ectD* gene which encodes the ectoine hydroxylase (Figure 2) (Bursy et al., 2007; Höppner et al., 2014; Widderich et al., 2014). Collectively, these results indicate that approximately 14% of the analyzed 163 genomes of *Planctomycetota* type strains harbor the genetic potential to synthesize ectoine, either alone (eight representatives), or in combination with hydroxyectoine (15 representatives).

Despite moderate overall sequence divergence, the key enzymes appear functionally conserved as indicated here for the signature enzymes for ectoine/hydroxyectoine biosynthesis, EctC and EctD. EctC-type proteins from *Planctomycetota* showed 47.8–69.2% amino acid identity to the EctC reference protein from *P. lautus* (Supplementary Figure S1) (Czech et al., 2019), while the amino acid sequence identity of EctD-type proteins ranged from 44.7–50.6% relative to the biochemically and structurally characterized ectoine hydroxylase from *Sphingopyxis alaskensis* (Supplementary Figure S2) (Höppner et al., 2014). Notably, residues critical for substrate binding and catalytic activity (Bursy et al., 2008; Richter et al., 2020; Czech et al., 2019; Höppner et al., 2014; Andrys-Olek et al., 2024; Widderich et al., 2014) were mostly preserved in the analyzed EctC and EctD proteins from *Planctomycetota* (Supplementary Figures S1, S2). Overall, our *in silico* analysis thus revealed that only a subset of *Planctomycetota* contained the genetic machinery necessary for ectoine and hydroxyectoine production (Czech et al., 2018; Hermann et al., 2020; Kunte et al., 2014; Pastor et al., 2010), potentially contributing to osmoadaptation in this phylum.

### 3.2 Phylogenomic distribution of ectoine biosynthetic gene clusters in type strains of *Planctomycetota*

**Figure 3:**
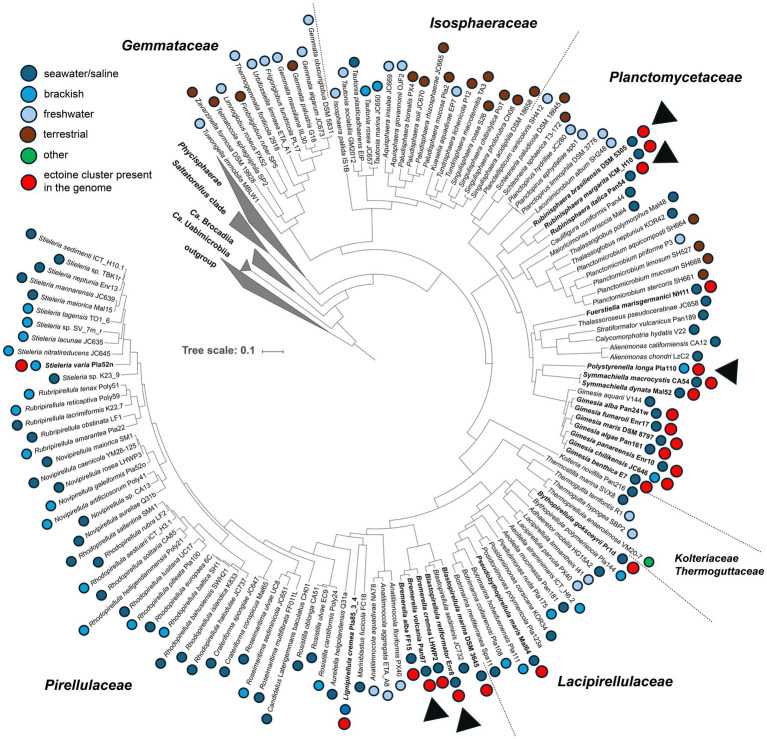
Multilocus sequence analysis (MLSA)-based phylogenetic tree. The phylogenetic tree was constructed based on the available reference genomes of the current 163 members of the phylum *Planctomycetota* available from the NCBI RefSeq database. Tree reconstruction was performed based on the 30 single copy gene-encoded proteins using autoMLST (Alanjary et al., 2019) with the outgroup described in the Material and methods section. Collapsed clades do not contain strains harboring ectoine biosynthetic gene clusters. All strains that harbor ectoine clusters are shown in bold and are highlighted with a red dot. Dots colored in green, brown, or different shades of blue indicate the type of habitat from which the strains have been isolated. Black triangles indicate strains that were selected for experimental validation of ectoine/hydroxyectoine biosynthesis or were used as a gene donor for heterologous expression of an *ectI*-type gene e in *Escherichia coli*. The information on the habitat from which the various type strains were originally isolated were obtained from their original description in the literature or from public databases (e.g., BacDive hosted by the DSMZ) (Schober et al., 2025).

To explore the phylogenetic distribution of predicted ectoine and hydroxyectoine producers (Figure 2) more closely, we constructed a multilocus sequence analysis (MSLA) based phylogenetic tree (Alanjary et al., 2019) encompassing the 163 analyzed reference genomes of the phylum *Planctomycetota* representing all currently know type strains (as of April 2026) (Figure 3). Labeling of strains harboring ectoine biosynthetic gene clusters in the tree revealed a skewed distribution of these genes, confined to specific subgroups within the falilies *Pirellulaceae*, *Planctomycetaceae* and L*acipirellulaceae*. Most *ect*-containing genomes were found among the *Planctomycetaceae* (14 out of 36 genomes) with three major clusters of predicted ectoine/hydroxyectoine producers formed (Figure 3). Notably, the majority of *Planctomycetaceae* (22 representatives) did not contain *ect* gene clusters and this is also true for members of the *Pirellulaceae* where only seven out of 56 representatives carry *ect* gene clusters (Figure 3). The *ectC*-containing genomes of *Pirellulaceae* are confined to a taxonomically closely related set of five *Blastopirellula* and *Bremerella* species (Figure 3). The *Pirellulaceae* contained in addition two representatives with *ect* gene clusters (*Lignipirellula cremea* Pla85_3_4 and *Stieleria* *varia* Pla52n) which appeared to be phylogenetically distant within the family (Figure 3). Likewise, of the 14 *Lacipirellulaceae*, only two genomes with *ect* gene clusters were identified (Figure 3).

Overall, these observations indicate that ectoine and hydroxyectoine biosynthesis in *Planctomycetota* is predominantly linage-specific and generally occurs only in three clearly separable major groups of species, each of which possesses close taxonomic relationships within the respective group (Figure 3). Taxonomically isolated representatives of the *Planctomycetota* with *ect* gene clusters might have acquired these gene through horizontal gene transfer, a major driver of microbial evolution (Van Etten and Johnson, 2026).

As recently observed (Czech et al., 2022; Gregory et al., 2020; Shikuma et al., 2013; Imhoff et al., 2020) many *ect* gene clusters co-localize with a gene for a MarR-type repressor (EctR/CosR) that is involved in regulating *ect* gene expression (Mustakhimov et al., 2010). With only four exceptions, the planctomycetotal *ect* gene clusters are associated with such a presumed regulatory gene (Figure 2). During comparison of planctomycetotal EctR proteins with the functionally characterized homologous protein from *Methylotuvimicrobium alcaliphilum* (Mustakhimov et al., 2010), a range of amino acid sequence identity of 24.7–45.9% was obtained (Supplementary Figure S3). The relatively high degree of *ectR* conservation in ectoine biosynthetic gene clusters in combination with the sequence homology of the only functionally studied EctR protein from *M. alcaliphilum* (Mustakhimov et al., 2010) suggests that EctR also serves as a transcriptional repressor for *ect* biosynthetic gene clusters in *Planctomycetota*. The gene for a specialized aspartokinase (*ask_ect*) (Stöveken et al., 2011) involved in precursor supply for ectoine biosynthesis (Figure 1) is often associated with *ect* biosynthetic gene clusters (Czech et al., 2018; Hermann et al., 2020), but it was not present in the vicinity of any planctomycetotal *ect* gene clusters. This indicates that these strains rely on canonical aspartokinases that catalyzes the initial step for the biosynthesis of L-lysine, L-threonine, L-isoleucine, L-methionine and the peptidoglycan precursor diaminopimelic acid (Lo et al., 2009).

### 3.3 Synthesis of ectoine/hydroxyectoine in selected *Planctomycetota* is responsive to salt stress

Compatible solutes such as ectoine and hydroxyectoine are well-known protectants against osmotic stress, and their synthesis is generally upregulated in response to either suddenly imposed or sustained increases in environmental osmolarity (Czech et al., 2018; Hermann et al., 2020; Pastor et al., 2010; Czech et al., 2018; Calderon et al., 2004). To evaluate whether the *in silico*–predicted ectoine/hydroxyectoine gene clusters in *Planctomycetota* are functional, we selected four representative species for experimental analyses: *R. italica* Pan54 (Kallscheuer et al., 2020) and *P. longa* Pla110 (Peeters et al., 2020), both from the family *Planctomycetaceae*, while *B. volcania* Pan97 (Rensink et al., 2020) and *B. retiformator* Enr8 (Kallscheuer et al., 2020) are members of the family *Pirellulaceae*. These four species thus represent the two major phylogenetic groups of predicted ectoine/hydroxyectoine producers among *Planctomycetota* (Figures 2, 3).

To assess ectoine/hydroxyectoine production in response to sustained osmotic stress, cells of the four strains were grown in chemically defined media under varying NaCl concentrations to impose different degrees of osmotic/saline-mediated stress onto the cells. Ectoine/hydroxyectoine accumulation in stationary phase cultures was measured using HPLC analysis (Czech et al., 2018; Kuhlmann and Bremer, 2002). Defined media were deliberately chosen for these experiments to prevent confounding effects arising from the uptake of pre-formed compatible solutes present in rich media, such as glycine betaine and proline from yeast extract (Thomas et al., 1994), or proline-containing peptides, which can be imported and hydrolyzed to release the compatible solute L-proline (Zaprasis et al., 2013). Importantly, uptake of exogenous osmotic stress protectants often suppresses the energetically costly *de novo* biosynthesis of compatible solutes (Hoffmann and Bremer, 2017; Oren, 1999). Hence, using defined media allowed us to directly assess the intrinsic biosynthetic capacity for ectoines of the chosen four planctomycetotal species.

**Figure 4:**
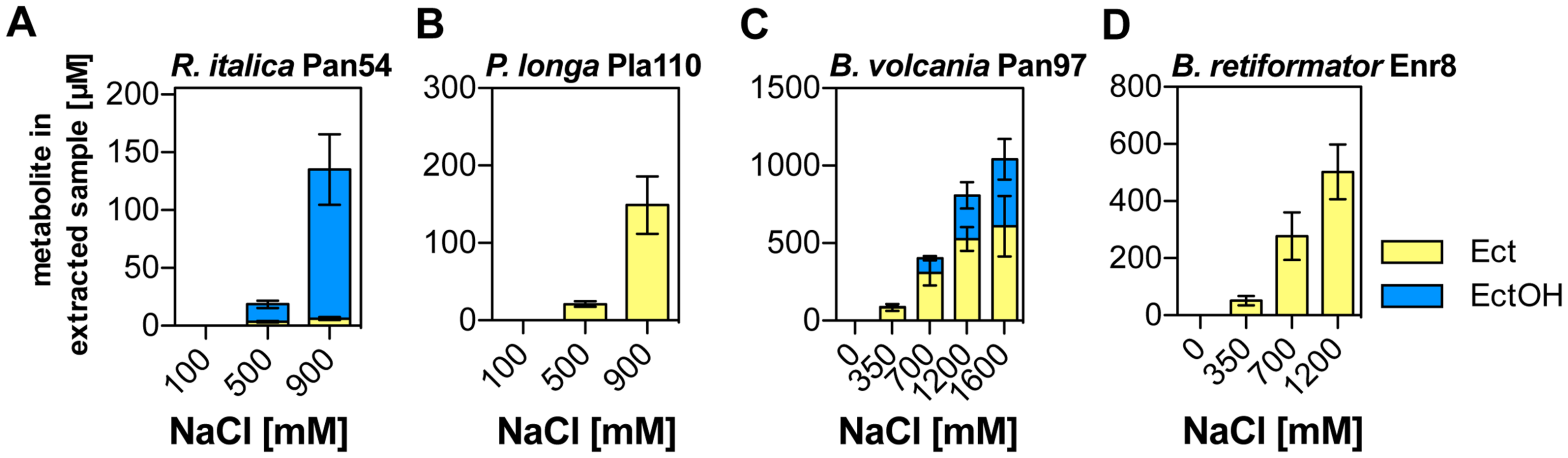
Intracellular ectoine and hydroxyectoine production in response to increasing salinity in selected *Planctomycetota*. Intracellular ectoine (Ect, yellow) and hydroxyectoine (EctOH, blue) were measured by HPLC analysis in **(A)** *Rubinisphaera italica* Pan54, **(B)** *Polystyrenella longa* Pla110, **(C)** *Bremerella volcanica* Pan97, and **(D)** *Blastopirellula retiformator* Enr8. *B. retiformator and B. volcania bel*ong to *Pirellulaceae*; *Rubinisphaera italica and Polystyrenella longa bel*ong to *Planctomycetaceae*. Strains were cultivated in chemically defined minimal media with either no added NaCl or increasing salt concentrations (as indicated) until the cultures reached stationary phase. Data represent the mean and standard error mean (SEM) of at least two biological replicates. Each biological replicate was analyzed in two technical replicates.

Inspection of the genome sequences suggested that *R. italica* Pan54 and *B. volcania* Pan97 should be capable of synthesizing both ectoine and hydroxyectoine, whereas *P. longa* Pla110 and *B. retiformator* Enr8 are predicted to produce exclusively ectoine (Figure 2). Experimental validation through growth assays and ectoine/hydroxyectoine quantification via HPLC analytics confirmed these *in silico* predictions. In all four species, the cellular content of ectoine and/or hydroxyectoine increased with rising NaCl concentrations in the growth medium (Figure 4), consistent with a role for these compounds as osmotic stress protectants (Czech et al., 2018; Hermann et al., 2020; Kunte et al., 2014; Pastor et al., 2010). As these measurements by HPLC analytics were conducted with cultures grown to stationary phase, they do not reflect the potential dynamics of ectoine and hydroxyectoine synthesis during different growth phases (Bursy et al., 2008; Kuhlmann and Bremer, 2002).

Although ectoine serves as the direct precursor for hydroxyectoine biosynthesis (Bursy et al., 2007), the production profiles for ectoines varied among the *ectABCD*-containing species *R. italica* Pan54 and *B. volcania* Pan97. *R. italica* Pan54 primarily accumulated hydroxyectoine, whereas *B. volcania* Pan97 produced both ectoine and hydroxyectoine, with their relative proportions shifting in response to the degree of salinity of the growth medium (Figures 4A,C). Although the enzymological and molecular basis underlying these differences in the pattern of ectoine/hydroxyectoine biosynthesis remains unclear, these findings highlight that, even among strains possessing the full ectoine/hydroxyectoine biosynthetic route (Figure 1), regulatory or enzymatic differences seemingly can influence the production ratios of these two compatible solutes in response to the environmentally imposed osmotic stress (Figures 4A,C).

### 3.4 The *ect* gene clusters of *Planctomycetota* are widely associated with presumptive importers for ectoines

Ectoine and hydroxyectoine biosynthetic gene clusters are often accompanied by genes encoding transport systems predicted to mediate the uptake of these compatible solutes (Hermann et al., 2020; Czech et al., 2022; Richter et al., 2019). Consistent with this general trend, genes for various types of transporters are positioned in the immediate genomic vicinity of all 23 ectoine/hydroxyectoine biosynthetic gene clusters identified in *Planctomycetota* (Figure 2). The encoded transporters belong to three major superfamilies: ATP-binding cassette (ABC) transporters (Davidson et al., 2008) such as the EhuABCD system from *Sinorhizobium meliloti* (Hanekop et al., *2007*; Jebbar et al., *2005*), the tripartite ATP-independent periplasmic (TRAP-T) systems (Mulligan et al., 2011) TeaABC from *Halomonas elongata* (Kuhlmann et al., 2008; Grammann et al., 2002) and UehABC from *Ruegeria pomeroyi* (Lecher et al., 2009; Schulz et al., *2017*), and members of the sodium solute symporter (SSS) family (Henriquez et al., 1880) such as EctI recently identified in *Hyphomonas neptunium* (Czech et al., 2022). This diversity of transporter types suggests that *Planctomycetota* have evolved multiple strategies to import ectoines, and possibly other types of compatible solutes (Czech et al., 2022), potentially reflecting sophisticated adaptation to distinct ecological niches or fluctuating osmotic conditions. Notably, no member of the BCCT family (Ziegler et al., 2010) [e.g., EctT (Kuhlmann et al., 2011)] or MFS-type transporters (Yan, 2015) [e.g., ProP (MacMillan et al., 1999)] was present; otherwise, these families of importers contain representative uptake systems for ectoine/hydroxyectoine acquisition (Czech et al., 2022; MacMillan et al., 1999; Kuhlmann et al., 2011; Vermeulen and Kunte, 2004).

Based on the type of transporter genes located adjacent to the *ect* biosynthetic cluster, we grouped planctomycetotal *ect* gene neighborhoods into three clades (Figure 2). Across the dataset, we identified eleven TeaABC/UehABC-type TRAP-T systems (Kuhlmann et al., 2008; Grammann et al., 2002), six EhuABCD-type ABC transporters (Hanekop et al., 2007; Jebbar et al., 2005), and six SSS-type (EctI) transporters (Czech et al., 2022). Accordingly, among the 23 transport systems identified, 17 are sodium-dependent transporters (TRAP-T and SSS-type) (Henriquez et al., 1880; Rosa et al., 2018), which may provide an advantage for microorganisms inhabiting ecological niches characterized by high salinity.

Interestingly, transporter types associated with the three major *ect* gene cluster within *Planctomycetota* taxa appear to co-vary with traits inherited from putative founder species. In one *ect*-containing *Planctomycetaceae* lineage comprising *R. brasiliensis* DSM 5305, *Rubinishaera margarita* ICM_H10, and *R. italica* Pan54 (Figure 3), *ect* clusters consistently co-occur with genes encoding EctI-type substrate-solute-symporters (SSS) (Czech et al., 2022) (Figure 2). By contrast, a second major *Planctomycetaceae ect* lineage predominantly encodes TRAP-type transporters related to the Tea and Ueh systems (Kuhlmann et al., 2008; Lecher et al., 2009), with the two *Lacipirellulaceae ect* representatives also sharing this feature. In *Pirellulaceae*, six of seven *ect*-containing genomes instead encode Ehu-type ABC transporters (Hanekop et al., 2007). The only exception, *Stieleria varia* Pla 52n, carries an EctI-type transporter and is taxonomically distinct from the main *ect*-containing *Pirellulaceae* cluster (Figure 3).

#### 3.4.1 *In silico* analysis of Ehu and tea/Ueh-type transporters for ectoines

Although mechanistically different (Davidson et al., 2008; Rosa et al., 2018), both ABC- and TRAP-type transporters depend on extracellular high-affinity substrate-binding proteins. TRAP-type transport systems function either both in osmotic stress protection and nutrient acquisition as shown for TeaABC from *Halomonas elongata* (Schwibbert et al., 2011; Kuhlmann et al., 2008), or solely in ectoine catabolism, as demonstrated for the amino acid sequence related UehABC transporter from *R. pomeroyi* (Lecher et al., 2009; Schulz et al., 2017). EhuABCD-type ABC transporters have similarly been linked to the import of ectoines either for stress protection or for nutritional purposes (Hanekop et al., 2007; Richter et al., 2019; Jebbar et al., 2005). Structural data for the TeaA, UehA, and EhuB substrate-binding proteins in complex with ectoines (Kuhlmann et al., 2008; Hanekop et al., 2007; Lecher et al., 2009) should therefore enable the functional annotation of transporter genes located adjacent to *ectABC(D)* biosynthetic loci in *Planctomycetota* (Figure 2).

Substrate-binding proteins from predicted EhuABCD-type and TeaABC-type transporters of *Planctomycetota* exhibited amino acid sequence identity (30.9–36.6%) to EhuB and TeaA from *S. meliloti* and *H. elongata*, respectively (Supplementary Figures S4–S7). Despite differences in physiological roles and transcriptional regulation (Grammann et al., 2002; Lecher et al., 2009), the ectoine/hydroxyectoine-specific periplasmic substrate-binding proteins of the TeaABC [Protein Data Base (PDB) entry 2VPN] and the UehABC [Protein Data Base (PDB) entry 3FXB] TRAP-transporter share nearly identical folds and binding-pocket architectures (Kuhlmann et al., 2008; Lecher et al., 2009). Comparable ectoine-binding sites are conserved in Tea/Ueh-related TRAP transporters, as well as in EhuB-type ABC transporter proteins (e.g., PDB 2Q88) (Hanekop et al., 2007) from *Planctomycetota* (Supplementary Figures S5, S6). These structural similarities strongly indicate that the genes encoding TRAP- and ABC-type transporter genes positioned adjacent to *ect* biosynthetic core genes in *Planctomycetota* (Figure 2) likely encode genuine ectoine/hydroxyectoine import systems.

#### 3.4.2 Functional assessment and substrate specificity of the EctI-type transporter from *Rubinisphaera brasiliensis*

Previous *in silico* structural modeling and ligand docking experiments (Czech et al., 2022) suggested that the SSS-type EctI transporter from *H. neptunium* adopts a evolutionary conserved LeuT fold similar to that of the SiaT N-acetylneuraminic acid transporter from *Proteus mirabilis* (Wahlgren et al., 2018). The predicted binding pocket for ectoine and hydroxyectoine in EctI (Czech et al., 2022) overlaps with the experimentally determined position of the N-acetylneuraminic acid ligand in the SiaT crystal structure [Protein Data Base (PDB) entry 5NVA] (Wahlgren et al., 2018). The six EctI-type proteins encoded by *Planctomycetota* (Figure 2) show amino acid sequence conservation with at least 38.1% sequence identity and a maximal value of 40.0% for EctI from *R. italica* Pan54 when compared with the *H. neptunium* EctI reference protein (Supplementary Figure S8).

Previous physiological studies demonstrated that *R. brasiliensis* DSM 5305 produces both ectoine and hydroxyectoine under hyperosmotic shock conditions (Ferreira et al., 2016). Consistent with these observations, our genomic inspection revealed that this species indeed carries an *ectABCD* biosynthetic gene cluster (Figure 2). It is flanked by a gene encoding an EctI-type transporter. The recently discovered ectoine/hydroxyectoine importer EctI from *H. neptunium* mediates uptake of ectoine (weak) and hydroxyectoine (efficient) but its substrate profile also comprises a range of other compatible solutes (Czech et al., 2022).

**Figure 5:**
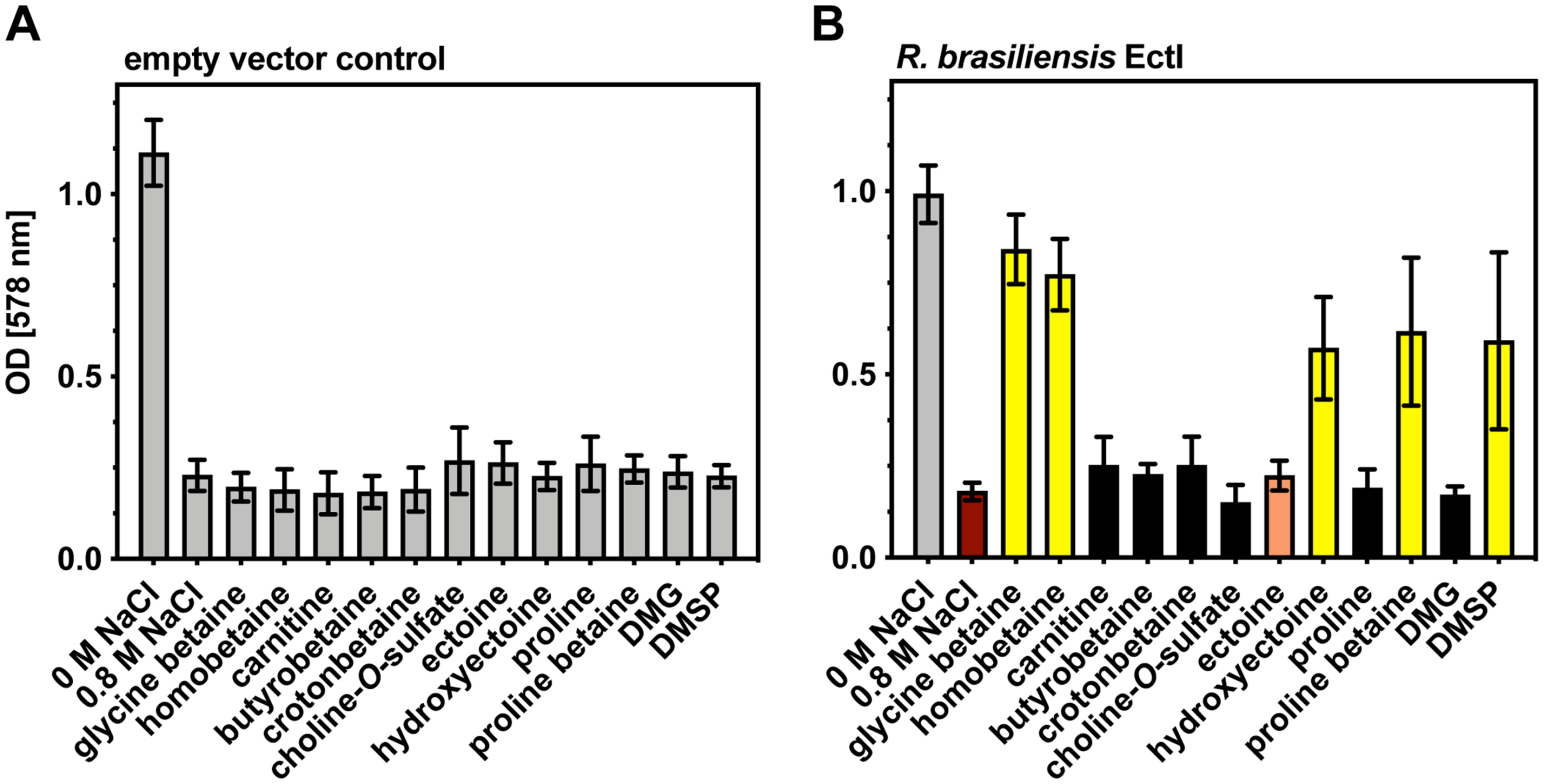
Osmotic stress protection and substrate specificity of the EctI transporter from *Rubinisphaera brasiliensis* DSM 5305. Growth yield (OD₅₇₈) of the *Escherichia coli str*ain MKH13, which lacks the compatible solute transporters ProP and ProU (Haardt et al., 1995; Lucht and Bremer, 1994; MacMillan et al., 1999) carrying either the empty vector pTrc99a (Amann et al., 1988) **(A)** or a pTrc99a-derived plasmid (pLC211) harboring a codon-optimized *ectI* gene from *Rubinisphera brasiliensis* (this study) **(B)**. The *ectI* gene present on plasmid pLC211 is expressed from an IPTG-inducible and LacI-responsive *lac* promoter present on the plasmid backbone of pTrc99a (Amann et al., 1988). Increased expression of *ectI was* induced with 0.3 mM IPTG and cultures were grown for 30 h at 37 °C in minimal medium (MMA) (Miller, 1972) without or with 0.8 M NaCl, supplemented with 1 mM of the indicated compatible solutes. Growth was monitored hourly; only final yields at 30 h are shown. Data represent mean ± SEM of at least four biological replicates.

Given that Ehu- and Tea/Ueh-type ectoine/hydroxyectoine transporters have been characterized in considerable detail (Kuhlmann et al., 2008; Hanekop et al., 2007; Richter et al., 2019; Jebbar et al., 2005; Grammann et al., 2002; Lecher et al., 2009), our study focused now on the EctI-type system from *R. brasiliensis* DSM 5305, especially because this strain has been experimentally confirmed to produce ectoine and hydroxyectoine (Ferreira et al., 2016). To this end, we performed a plasmid-borne inducible expression of a codon-optimized *R. brasiliensis* DSM 5305 *ectI* gene in the *E. coli* tester strain MKH13. This expression host lacks all known compatible solute uptake systems (BetT, ProP, ProU) (Haardt et al., 1995) and therefore only grows efficiently under high-salinity conditions (minimal medium containing 0.8 M NaCl) when a heterologous compatible solute importer gene is expressed and the cognate substrate is supplied to the growth medium (Czech et al., 2022; Kempf and Bremer, 1995). Accordingly, we subjected the recombinant MKH13 strain to osmotic stress protection assays in the presence of twelve different compatible solutes. The *R. brasiliensis* DSM 5305 EctI transporter supported robust growth under osmotic stress when the compatible solutes hydroxyectoine, glycine betaine, homobetaine, proline betaine, or DMSP were present in the medium (Figure 5). This substrate range closely mirrors the profile previously reported for the *H. neptunium* EctI transporter (Czech et al., 2022). Although limited in scope, these results indicate that *Planctomycetota* EctI transporters share functional properties with their counterpart from the marine bacterium *H. neptunium*.

Protection against osmotic stress via ectoine accumulation by the *R. brasiliensis* DSM 5305 EctI system was surprisingly weak (Figure 5), recapitulating similar findings in the *E. coli* strain MKH13 expressing the *H. neptunium* EctI transporter (Czech et al., 2022). Hydroxyectoine, efficiently imported by both transporters (Figure 5) (Czech et al., 2022), likely shares ligand-binding determinants overlapping with those for ectoine, as seen in the crystal structures of the substrate-binding proteins form the TeaABC and EhuABCD complexed with either ectoine or hydroxyectoine (Kuhlmann et al., 2008; Hanekop et al., 2007). Considering the chemical similarity between the ectoine and hydroxyectoine molecules (Figure 1), the unexpectedly poor uptake of ectoine by EctI from *H. neptunium* (Czech et al., 2022) and *R. brasiliensis* DSM 5305 (Figure 5) thus exhibits a puzzling substrate profile that remains to be studied further. We currently have no suitable explanation for this phenomenon.

## 4 Discussion

Members of the *Planctomycetota*, a phylum recognized for its ecological versatility, unusual cell biology, and biotechnological potential (Kündgen et al., 2025; Wiegand et al., 2018; Kallscheuer and Jogler, 2021; Lage et al., 2025; Hägglund et al., 2026), synthesizes a variety of compatible solutes in response to osmotic stress (d'Avo et al., 2013; Cunha et al., 2013; Ferreira et al., 2016; Schwibbert et al., 2011; Jeske et al., 2013; Wecker et al., 2009). The extremolytes ectoine and hydroxyectoine are widely used by bacteria to tolerate hyperosmotic stress and extreme growth temperatures (Czech et al., 2018; Hermann et al., 2020; Kunte et al., 2014; Pastor et al., 2010; Imhoff et al., 2020; Reshetnikov et al., 2011; Gregory and Boyd, 2021). Although these compatible solutes have previously been experimentally detected in *G. maris* and *R. brasiliensis* (Ferreira et al., 2016), their broader occurrence across the *Planctomycetota* remains poorly understood.

Using a phylogenomic framework, we examined the distribution, potential evolutionary history, and functionality of ectoine/hydroxyectoine biosynthetic gene clusters across all 163 currently available type strain genomes of the phylum *Planctomycetota* (Wiegand et al., 2018; Lage et al., 2025; Devos et al., 2020). By prioritizing type strains over rapidly expanding metagenomic datasets, we ensured analytical rigor while maintaining access to cultivable reference isolates for downstream experimental validation and follow-up studies. Our analyses revealed that approximately 14% of the currently described type strains with available genomes harbor *ect* biosynthetic genes (Figure 2). Notably, the biosynthetic capacity for these extremolytes is distributed in a lineage-specific manner across the phylum and is largely restricted to the *Planctomycetaceae* and *Pirellulaceae* families, with sporadic occurrence in the *Lacipirellulaceae* (Figure 3). The occurrence of *ect* biosynthetic genes in species that cluster together, suggests their acquisition by ancestral founder species followed by lineage-specific diversification of individual genera.

This lineage-specific diversification is also observable by the types of presumed ectoine/hydroxyectoine transport systems whose structural genes co-localize with the *ect* core biosynthetic genes (Figure 2). Such genomic arrangements may indicate that ancestral founder species repeatedly acquired the capacity for ectoine biosynthesis and transport through independent horizontal gene transfer events, thereby potentially contributing to evolutionary adaptation and physiological innovation to counteract the detrimental effects of high osmolarity/salinity on cellular physiology and growth (Bremer and Krämer, 2019; Czech et al., 2018).

Gene gain through horizontal gene transfer is a well-established driver of bacterial evolution and innovation (Van Etten and Johnson, 2026), and appears to have also shaped one of the most important physiological traits of *Planctomycetota*, anaerobic ammonium oxidation (anammox) (Bengtsson and Ovreas, 2010). Operons and functionally associated gene clusters are uncommon in *Planctomycetota*, with even related genes often dispersed throughout the genome. Therefore, the occurrence of clustered ectoine and hydroxyectoine biosynthesis and transport genes in this phylum (Figure 2) is particularly noteworthy. In the context of osmotic stress adaptation, genes encoding the L-proline transporter OpuE and a glycine/sarcosine N-methyltransferase involved in glycine betaine biosynthesis appear to have been acquired through horizontal gene transfer in *Planctomycetota* from hypersaline microbial mats (Skoog et al., 2023). Likewise, studies of ectoine-producing methylotrophs, some *Archaea*, and marine members of the *Rhodobacteraceae* suggest that acquisition of ectoine biosynthesis genes through horizontal gene transfer has contributed to environmental adaptation across diverse microbial lineages (Reshetnikov et al., 2011; Widderich et al., 2016; Simon et al., 2017).

Notably, all 23 *Planctomycetota* species capable of ectoine/hydroxyectoine biosynthesis were originally isolated from seawater or saline habitats (Figure 3), environments that impose sustained osmotic stress due to seawater salinity (~35 g salt per kg water). Accordingly, ectoine synthesis and uptake are expected to support physiological adaptation to the persistent osmotic stress of marine, saline and brackish environments (Czech et al., 2018; Kunte et al., 2014; Pastor et al., 2010). However, not all *Planctomycetota* isolated from high-salinity habitats possess *ect* biosynthetic genes; in fact, most saline-associated species lack these genes (Figure 2). These organisms are therefore likely to rely on alternative compatible solutes for osmotic stress protection, as exemplified by the biosynthesis of a cocktail of compatible solutes (glutamate, glucosylglycerate, mannosyl-(1,2)-glucosylglycerate, trehalose) in the marine species *R. baltica* (d'Avo et al., 2013; Cunha et al., 2013).

Among compatible solutes, ectoines have attracted considerable commercial interest due to their cytoprotective and chemical chaperone properties (Kunte et al., 2014; Pastor et al., 2010; Becker and Wittmann, 2020; Kadam et al., 2024). Hydroxyectoine has higher commercial value than ectoine due to broader cytoprotective effects (Knapp et al., 1999; Tanne et al., 2014; Bursy et al., 2008; Argandona et al., 2021; Manzanera et al., 2004). Accordingly, various microbial production hosts are currently being explored to improve fermentative yields of hydroxyectoine. Hydroxyectoine is more difficult to isolate in pure form than ectoine because its synthesis depends on prior ectoine formation (Bursy et al., 2007) causing many producing microorganisms to generate both compounds as mixtures, as observed here in the salt-stress-dependent production profile of *B. volcania* Pan97 (Figure 4C). *R. italica* Pan54 notably deviates from this pattern, producing almost exclusively hydroxyectoine (Figure 4A). As *Planctomycetota* gain attention as cell factories for the biotechnological production of bioactive small molecules (Kallscheuer and Jogler, 2021), selected representatives of this phylum may be exploitable as natural production hosts for pure ectoine and hydroxyectoine.

Across microorganisms, *ect* biosynthetic genes are frequently linked to genes encoding various ectoine/hydroxyectoine transport systems (Czech et al., 2022; Richter et al., 2019). This pattern can also be observed in *Planctomycetota*, where all 23 *ect*-containing species possess putative ectoine/hydroxyectoine importer genes (Figure 2). This genetic association may indicate a selective advantage in osmotically challenging environments, enabling coordination of ectoine production with uptake (Czech et al., 2018; Czech et al., 2022). Ectoines and other compatible solutes are released into the environment by producer organisms through cell lysis, active export, or transient opening of mechanosensitive channels during osmotic downshifts (Czech et al., 2022; Czech et al., 2016; Hobmeier et al., 2022; Warren, 2013; Welsh, 2000).

In general, the import of these solutes under osmotically challenging conditions benefits not only ectoine/hydroxyectoine-producing cells but also species that lack biosynthetic capacity and rely solely on their uptake (Bremer and Krämer, 2019; Czech et al., 2018; Czech et al., 2022; Wood et al., 2001). The energetic advantage of importing ectoine instead of synthesizing it is dramatic. When cells take up ectoine using an ABC transporter such as EhuABCD (Hanekop et al., 2007; Richter et al., 2019; Jebbar et al., 2005), only two ATP molecules are consumed per imported ectoine molecule (Davidson et al., 2008). In contrast, producing a single ectoine molecule from scratch requires approximately 40 ATP equivalents (Oren, 1999; Oren, 2011). Thus, ectoine uptake via an ABC transporter consumes only approximately one-twentieth of the energy required for biosynthesis. Other classes of compatible solute transporters (Czech et al., 2022) are likewise anticipated to also provide energetic benefits to cells already challenged by energy constraints (Bremer and Krämer, 2019; Wood, 2011; Oren, 1999; Oren, 2011). Furthermore, the import of compatible solutes often suppresses the energetically costly *de novo* synthesis of extremolytes (Bremer and Krämer, 2019; Hoffmann and Bremer, 2017; Czech et al., 2018).

*Planctomycetota* possessing both *ect* biosynthetic genes and ectoine transport systems (Figure 2) can synthesize ectoines when these compatible solutes are absent from their ecological niche and import them when they are available. This dual capability enables flexible, energy-efficient regulation and fine-tuning of the osmotic stress-responsive extremolyte pool. Such adaptability is particularly advantageous in dynamic environments characterized by fluctuations in salinity and water availability. Furthermore, many *Planctomycetota* form biofilms, where extracellular ectoines, like the role of glycine betaine in *Vibrio cholerae* biofilms (Kapfhammer et al., 2005), may serve as communal osmoprotectants, with transport systems playing central roles in their uptake and distribution. Supporting this concept, transcriptional analyses of *Novosphingobium* sp. LH128 (Czech et al., 2022) revealed strong upregulation of both *ect* biosynthetic and transporter genes in biofilms exposed to acute and prolonged high-osmolarity conditions (Fida et al., 2012).

## Data Availability

The original contributions presented in the study are included in the article/Supplementary material, further inquiries can be directed to the corresponding author/s.
