# Supplementary material for: Biosynthesis and import of the cytoprotective extremolytes ectoine and hydroxyectoine in the phylum Planctomycetota
Source: Front Microbiol. 2026 Jun 24;17:1823765. doi: 10.3389/fmicb.2026.1823765 (PMC13342039; doi:10.3389/fmicb.2026.1823765)
Supplement: Supplementary file 1 [file Data_sheet_1.pdf]

Supplementary Material

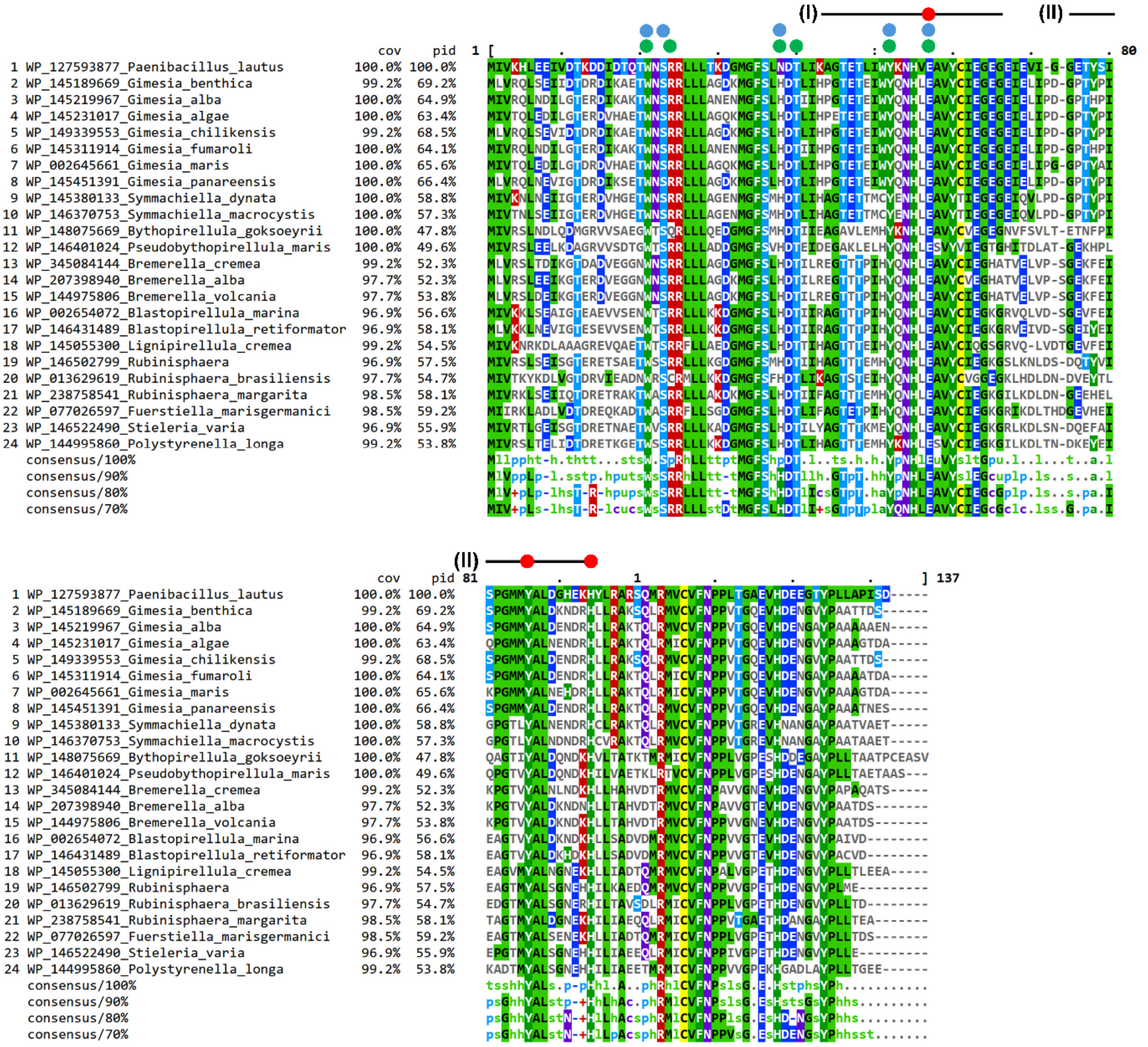

Figure S1. Alignment of EctC proteins found in type strains of described members of the phylum *Planctomycetota* in comparison to the EctC synthase from *P. lautus*. The amino acid sequence identities for the ectoine synthase (EctC), the signature enzyme of the ectoine biosynthetic route [1], range between 47.8% (type strain of *Bythopirellula goksoeyrii*) for and 69.2% for the type strain of *Gimesia benthica* in comparison to the biochemically and structurally characterized *P. lautus* EctC protein [2]. NCBI protein accession numbers are provided for each sequence. Residues crucial for binding of the iron catalyst, the reaction product ectoine and the substrate N-γ-ADABA by the *P. lautus* EctC enzyme [2] are marked with a red, blue and green dot, respectively.

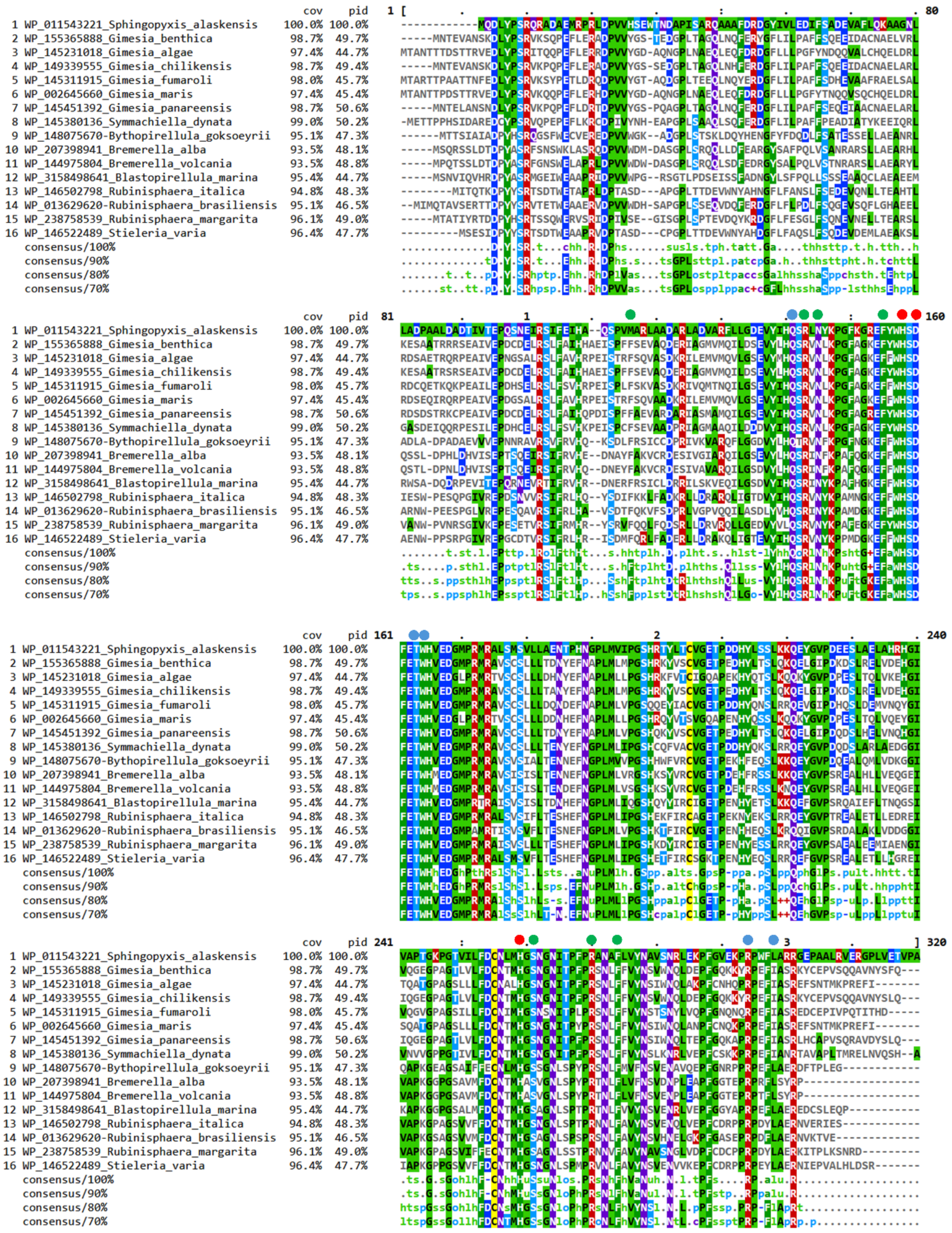

Figure S2. Alignment of EctD proteins found in *Planctomycetes* in comparison to the EctD hydroxylase from *S. alaskensis*. Amino acid sequence identities of ectoine hydroxylases (EctD) range between 44.7% (*Gimesia algae* and *Blastopirellula marina*) to 50.6% (*Symmachiella dynata*) in comparison to the biochemically and structurally characterized EctD protein from *S. alaskensis* [3]. The type of *Symmachiella macrocystis* also harbors an *ectD* gene, but the open reading frame is interrupted by a nonsense mutation. NCBI protein accession numbers are provided for each sequence. Residues crucial for binding of the iron catalyst, the reaction product 5-hydroxyectoine and the co-substrate 2-oxoglutarate in *S. alaskensis* EctD are marked with a red, blue and green dot, respectively.

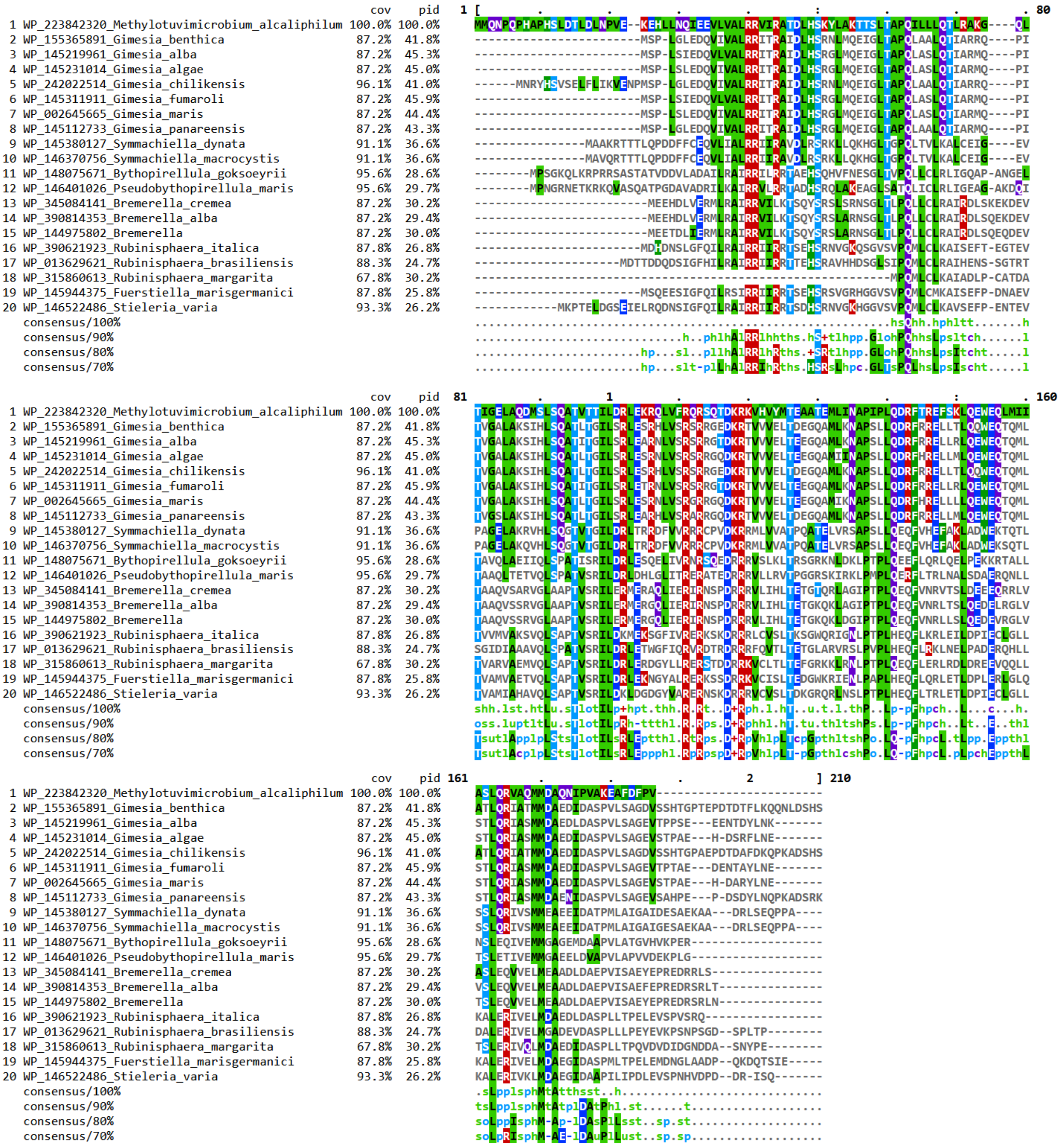

Figure S3. Alignment of the protein sequences of the MarR-type transcriptional regulator EctR compared with the homologous protein from *M. alcaliphilum*. Amino acid sequence identities of *Planctomycetota* EctR proteins range between 24.7% (*Rubinishaera brasiliensis*) and 45.9% (*Gimesia fu*maroli) compared to the biochemically and structurally studied protein from *M. alcaliphilum* [4]. NCBI protein accession numbers are provided for each sequence.

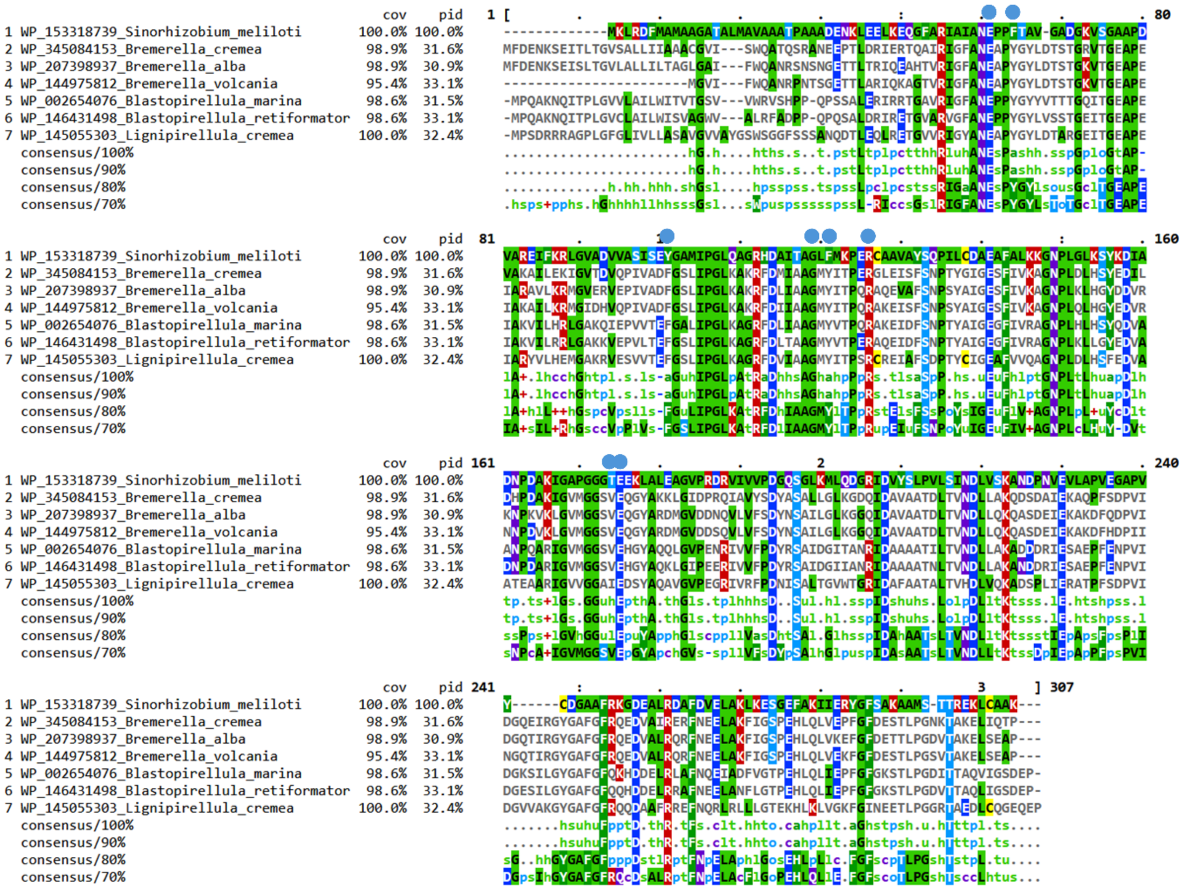

Figure S4. Alignment of EhuB-type substrate-binding proteins from the ectoine/ hydroxyectoine ABC-type transporter EhuABCD, located in the gene neighborhoods of ectoine biosynthetic gene clusters in *Planctomycetota*, compared with EhuB from *S. meliloti*. Amino acid sequence identities of *Planctomycetota* EhuB-type proteins range between 30.9% for the type strain of *Bremerella alba* and 33.1% for the type strains of *Bremerella volcania* and *Blastopirellula retiformator* compared to the biochemically and structurally studied EhuB protein from *S. meliloti* [5]. NCBI protein accession numbers are provided for each sequence. Residues crucial for ligand-binding of ectoine/5-hydroxyectoine in the *S. meliloti* EhuB substrate binding protein are marked with a blue dot.

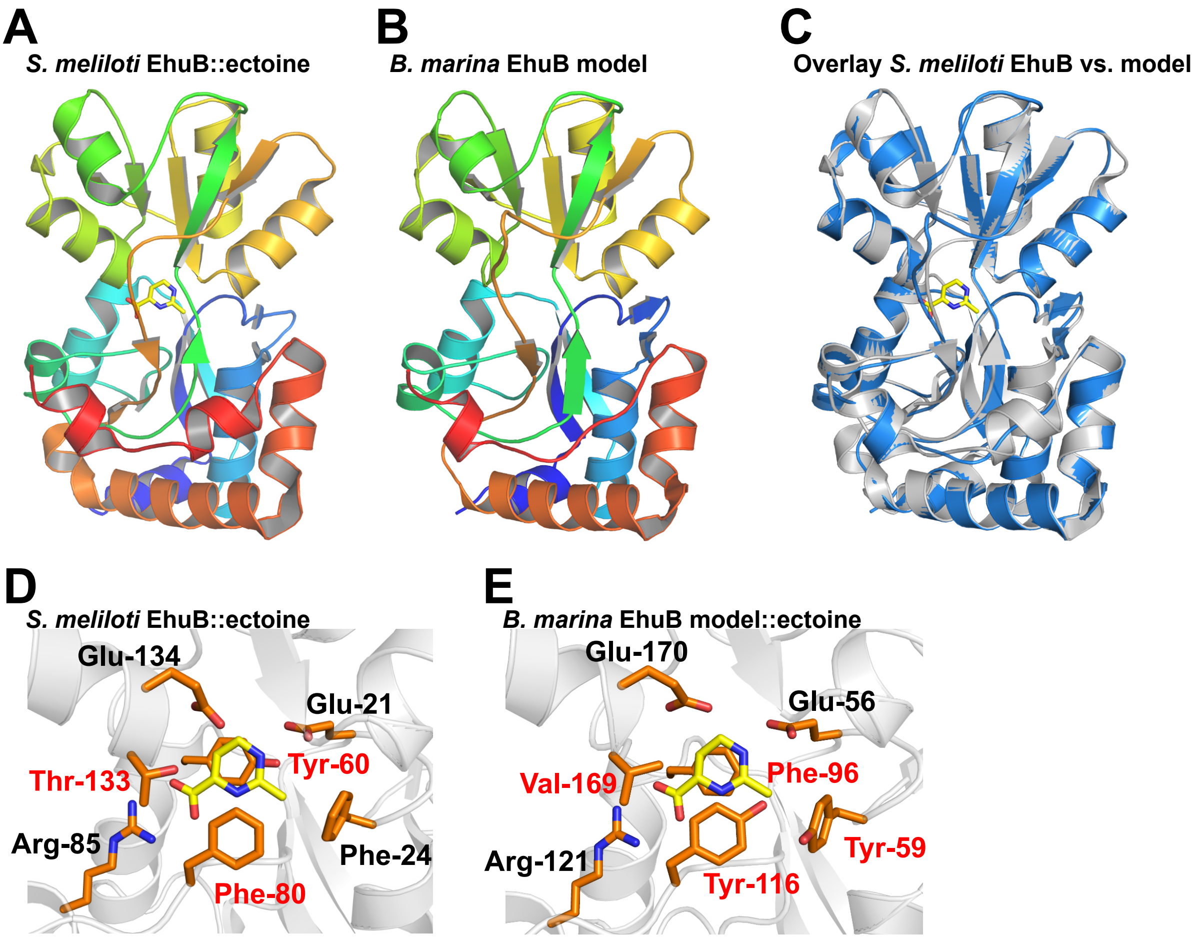

Figure S5. Model of the EhuB-type substrate-binding protein from *Blastopirellula marina* DSM 3645 compared with the experimentally determined crystal structure of the *Sinorhizobium meliloti* EhuB protein in complex with ectoine. The crystal structure of the periplasmic EhuB substrate binding protein has been determined in complex with its ligand ectoine (Protein Data Bank accession code 2Q88) [5]. The EhuB protein can also bind hydroxyectoine. It belongs to a gene cluster involved in the import and catabolism of ectoines when they are used as nutrients [6] but genes for EhuABCD-type ABC-systems have also been identified in the context of osmotic stress-adaptive ectoine/hydroxyectoine biosynthetic gene clusters [7]. **(A-C)** Overall fold of EhuB in complex with ectoine (represented in yellow sticks) compared to an *in silico* model of the *B. marina* EhuB-type protein. The amino acid side chains highlighted in red in (**D**) and (**E**) differ between the *S. meliloti* and *B. marina* EhuB-type proteins; these variations arise mostly from substitutions of functionally equivalent residues (e.g., Tyr-60 in *S. meliloti* replaced by Phe-96 in the *B. marina* EhuB protein).

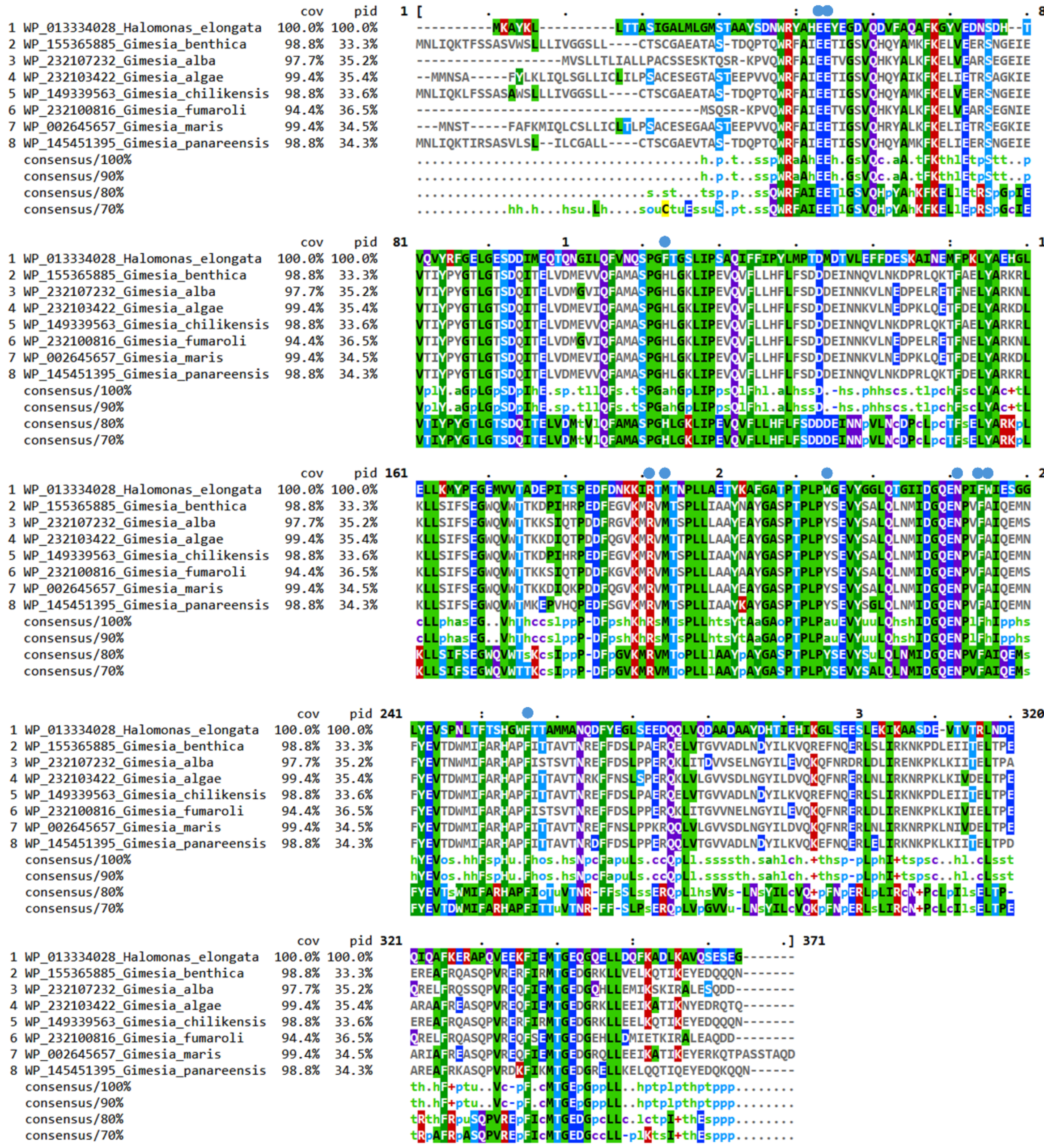

Figure S6. Alignment of TeaA-type substrate-binding proteins from the ectoine transporter TeaABC in type strain of species belonging to the phylum *Planctomycetota*, compared to the TeaA substrate-binding protein from *H. elongata*. Amino acid sequence identities of periplasmic TeaA-type proteins from described *Planctomycetota* range between 33.3% (*Gimesia benthica)* and 36.5% (*Gimesia fumaroli*) compared to the biochemically and structurally characterized TeaA protein from *H. elongata* [8; 9]. NCBI protein accession numbers are provided for each sequence. Residues crucial for binding of ectoine/5-hydroxyectoine in the *H. elongata* TeaA protein are marked with a blue dot.

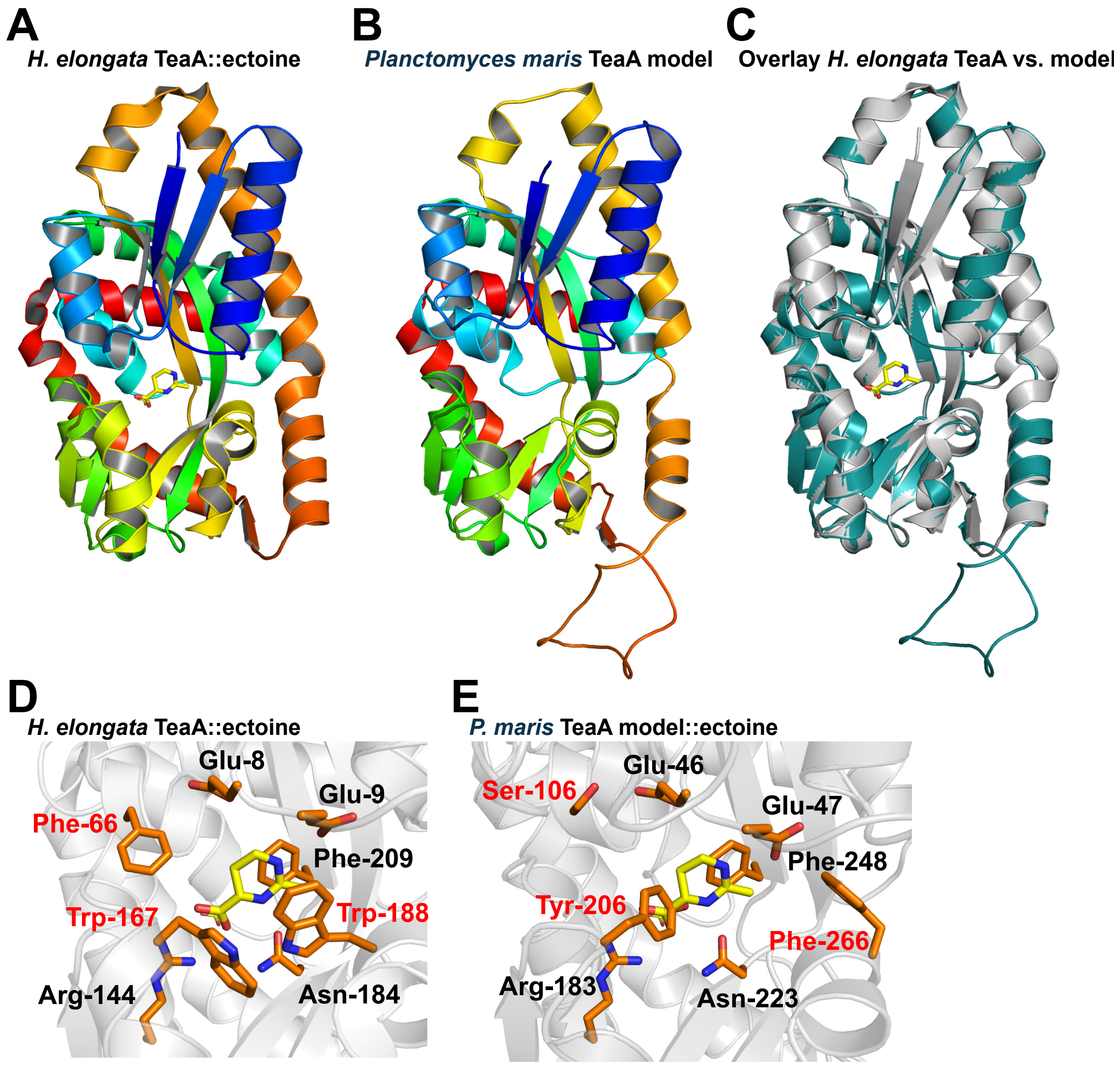

Figure S7. Structural model of the EhuB-type substrate-binding protein from *Gimesia maris* DSM 8797 (basonym: *Planctomyces maris*) compared with the crystal structure of *Halomonas elongata* TeaA bound to ectoine. The crystal structure of the periplasmic TeaA substrate binding protein from *H. elongata* has been determined in complex with its ligand ectoine (represented in yellow sticks) (Protein Data Bank accession code 2Q88) [9]. The TeaA protein can also bind hydroxyectoine [9]. **(A-C)** Overall fold of TeaA in complex with ectoine compared to an *in silico* model of the *B. maris* TeaA-type substrate binding protein. The amino acid side chains highlighted in red in (**D**) and (**E**) differ between the *H. elongata* and *G. maris* TeaA-type; these variations arise mostly from substitutions of functionally equivalent residues (e.g., Trp-188 in *H. elongata* replaced by Phe-266 in the *G. maris* TeaA protein).

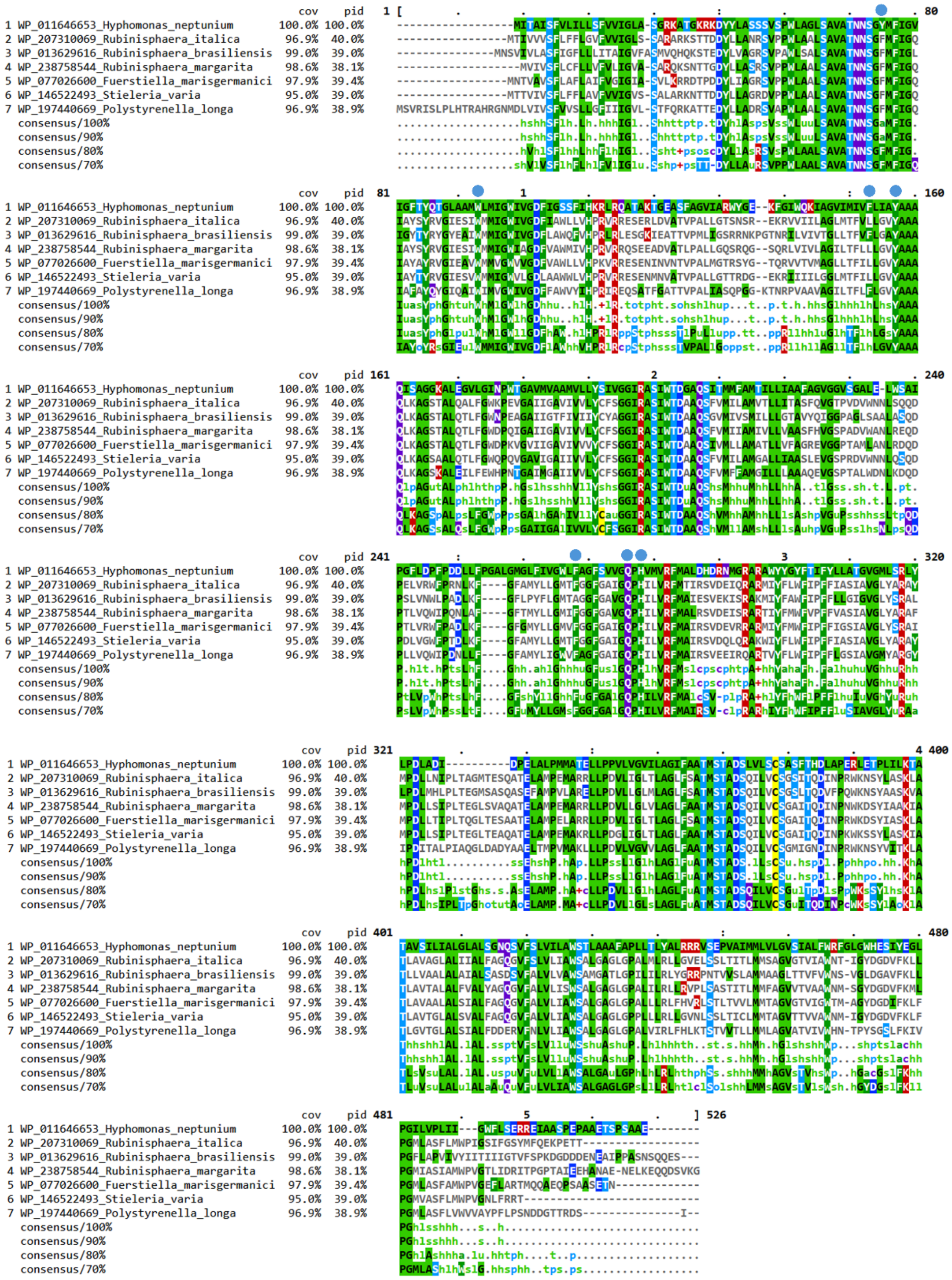

Figure S8. Alignment of EctI-type transporter proteins from *Planctomycetota* ectoine biosynthetic gene neighborhoods, compared to EctI from *H. neptunium*. Amino acid sequence identities of EctI-type transporters range between 38.1% (*Rubinisphaera margarita*) and 40.0% for (*Rubinisphaera italica*) compared to the EctI protein from *H. neptunium* [10]. The overall fold of EctI-type proteins resembles that of the *Proteus mirabilis* SiaT transporter for sialic acids [11]. Residues predicted *in silico* to be involved in the binding of ectoine/5-hydroxyectoine in the *H. neptunium* EctI protein [10] are marked with a blue dot. The experimentally determined position of the sialic acid binding site in SiaT and the *in silico* predicted binding site for ectoines seem to overlap [10; 11].

REFERENCES:

[1] L. Hermann, C.N. Mais, L. Czech, S.H.J. Smits, G. Bange, and E. Bremer, The ups and downs of ectoine: structural enzymology of a major microbial stress protectant and versatile nutrient. Biol Chem 401 (2020) 1443-1468.

[2] L. Czech, A. Höppner, S. Kobus, A. Seubert, R. Riclea, J.S. Dickschat, J. Heider, S.H.J. Smits, and E. Bremer, Illuminating the catalytic core of ectoine synthase through structural and biochemical analysis. Sci Rep 9 (2019) 364.

[3] A. Höppner, N. Widderich, M. Lenders, E. Bremer, and S.H.J. Smits, Crystal structure of the ectoine hydroxylase, a snapshot of the active site. J Biol Chem 289 (2014) 29570-29583.

[4] Mustakhimov, II, A.S. Reshetnikov, A.S. Glukhov, V.N. Khmelenina, M.G. Kalyuzhnaya, and Y.A. Trotsenko, Identification and characterization of EctR1, a new transcriptional regulator of the ectoine biosynthesis genes in the halotolerant methanotroph *Methylomicrobium alcaliphilum* 20Z. J Bacteriol 192 (2010) 410-417.

[5] N. Hanekop, M. Höing, L. Sohn-Bösser, M. Jebbar, L. Schmitt, and E. Bremer, Crystal structure of the ligand-binding protein EhuB from *Sinorhizobium meliloti* reveals substrate recognition of the compatible solutes ectoine and hydroxyectoine. J Mol Biol 374 (2007) 1237-1250.

[6] M. Jebbar, L. Sohn-Bösser, E. Bremer, T. Bernard, and C. Blanco, Ectoine-induced proteins in *Sinorhizobium meliloti* include an ectoine ABC-type transporter involved in osmoprotection and ectoine catabolism. J Bacteriol 187 (2005) 1293-1304.

[7] A.A. Richter, C.N. Mais, L. Czech, K. Geyer, A. Hoeppner, S.H.J. Smits, T.J. Erb, G. Bange, and E. Bremer, Biosynthesis of the stress-protectant and chemical chaperon ectoine: biochemistry of the transaminase EctB. Front Microbiol 10 (2019) 2811.

[8] K. Grammann, A. Volke, and H.J. Kunte, New type of osmoregulated solute transporter identified in halophilic members of the bacteria domain: TRAP transporter TeaABC mediates uptake of ectoine and hydroxyectoine in *Halomonas elongata* DSM 2581(T). J Bacteriol 184 (2002) 3078-3085.

[9] S.I. Kuhlmann, A.C. Terwisscha van Scheltinga, R. Bienert, H.J. Kunte, and C. Ziegler, 1.55 A structure of the ectoine binding protein TeaA of the osmoregulated TRAP-transporter TeaABC from *Halomonas elongata*. Biochemistry 47 (2008) 9475-9485.

[10] L. Czech, C. Gertzen, S.H.J. Smits, and E. Bremer, Guilty by association: importers, exporters and MscS-type mechanosensitive channels encoded in biosynthetic gene clusters for the stress-protectant ectoine. Environ Microbiol 24 (2022) 5306-5331.

[11] W.Y. Wahlgren, E. Dunevall, R.A. North, A. Paz, M. Scalise, P. Bisignano, J. Bengtsson-Palme, P. Goyal, E. Claesson, R. Caing-Carlsson, R. Andersson, K. Beis, U.J. Nilsson, A. Farewell, L. Pochini, C. Indiveri, M. Grabe, R.C.J. Dobson, J. Abramson, S. Ramaswamy, and R. Friemann, Substrate-bound outward-open structure of a Na(+)-coupled sialic acid symporter reveals a new Na(+) site. Nature communications 9 (2018) 1753.
